# Endothelial Jagged1 promotes solid tumor growth through both pro-angiogenic and angiocrine functions

**DOI:** 10.18632/oncotarget.4380

**Published:** 2015-06-08

**Authors:** Ana-Rita Pedrosa, Alexandre Trindade, Catarina Carvalho, José Graça, Sandra Carvalho, Maria C. Peleteiro, Ralf H. Adams, António Duarte

**Affiliations:** ^1^ Centro Interdisciplinar de Investigação em Sanidade Animal (CIISA), University of Lisbon, Lisbon, Portugal; ^2^ Instituto Gulbenkian de Ciência, Oeiras, Portugal; ^3^ Department of Tissue Morphogenesis, Max Planck Institute for Molecular Biomedicine, Muenster, Germany; ^4^ Faculty of Medicine, University of Muenster, Muenster, Germany

**Keywords:** Jagged1, Notch, TRAMP, tumor angiogenesis, angiocrine

## Abstract

Angiogenesis is an essential process required for tumor growth and progression. The Notch signaling pathway has been identified as a key regulator of the neo-angiogenic process. Jagged-1 (*Jag1*) is a Notch ligand required for embryonic and retinal vascular development, which direct contribution to the regulation of tumor angiogenesis remains to be fully characterized.

The current study addresses the role of endothelial Jagged1-mediated Notch signaling in the context of tumoral angiogenesis in two different mouse tumor models: subcutaneous Lewis Lung Carcinoma (LLC) tumor transplants and the autochthonous Transgenic Adenocarcinoma of the Mouse Prostate (TRAMP).

The role of endothelial Jagged1 in tumor growth and neo-angiogenesis was investigated with endothelial-specific *Jag1* gain- and loss-of-function mouse mutants (*eJag1*OE and *eJag1c*KO). By modulating levels of endothelial *Jag1*, we observed that this ligand regulates tumor vessel density, branching, and perivascular maturation, thus affecting tumor vascular perfusion. The pro-angiogenic function is exerted by its ability to positively regulate levels of *Vegfr-2* while negatively regulating *Vegfr-1*. Additionally, endothelial Jagged1 appears to exert an angiocrine function possibly by activating Notch3/Hey1 in tumor cells, promoting proliferation, survival and epithelial-to-mesenchymal transition (EMT), potentiating tumor development. These findings provide valuable mechanistic insights into the role of endothelial Jagged1 in promoting solid tumor development and support the notion that it may constitute a promising target for cancer therapy.

## INTRODUCTION

Since Folkman's seminal insight of treating cancer by cutting its blood supply [1] much effort has been devoted to understand the underlying molecular mechanisms that drive tumor angiogenesis. It is well established that tumor growth is restricted in an early avascular phase, and that, to be able to progress and develop it requires an angiogenic switch [2].

Tumor angiogenesis is initiated when endothelial cells respond to local stimuli and migrate towards the growing mass. This migration results in the formation of tubular structures that ultimately recruit perivascular support cells in order to create a well-established neo-vasculature that allows tumor development and eventual metastization [2].

Many signaling pathways have been identified as key contributors to the neo-angiogenic process. Among them is the Notch signaling pathway, an evolutionary conserved signaling system that regulates proliferation, differentiation, cell-fate determination, progenitor and stem-cell self-renewal, in both embryonic and adult tissues [3, 4]. The Notch pathway is composed of 5 ligands (Jagged-1, Jagged-2, and Delta-like 1, 3, and 4) and 4 receptors (Notch 1–4). Ligand–receptor interactions promote the cleavage of the Notch receptors, releasing the Notch intracellular domain (NICD), which is then translocated to the nucleus where it binds a transcriptional repressor and ultimately leads to the transcription of downstream target genes, such as several helix–loop–helix transcription factors (*Hey* and *Hes* gene families among others) [3].

The Notch ligand, Dll4, is required for normal arterial patterning in the embryo [4] and has a major effect in solid tumor growth [5, 6]. This effect of targeting Dll4 is apparently paradoxical as it inhibits tumor growth by triggering excessive angiogenesis, that results in poorly functional vessels [7].

However, despite the extensive characterization of the role of Dll4 in tumor vasculature, the contribution of other Notch ligands, like Jagged1, is less well studied. *Jag1*-null mouse mutants die at E11.5 due to heart defects and abnormal development of the yolk sac and head vasculature [8]. Moreover, mutations in the human *JAG1* gene cause Alagille syndrome, which comprises complex cardiac defects and vascular anomalies [9]. Additionally, in the developing retina [10] endothelial Jagged1 has been shown to have a pro-angiogenic function, opposite to that of Dll4. This pro-angiogenic function has also been demonstrated in an adult physiological setting, where it promotes wound healing by the ability to antagonize Dll4/Notch1 endothelial branching while positively regulating vascular maturation through activation of endothelial Notch4 and perivascular Notch3 [11]. Jagged1 is expressed in the vasculature, as well as in many other tissues. In the context of tumor angiogenesis two reports suggest that tumor cells expressing Jagged1 can act in a pro-angiogenic manner: induction of the Notch ligand Jagged1 by growth factors (via MAPK) in head and neck squamous cell carcinoma was shown to trigger Notch activation in neighboring endothelial cells and promote capillary-like sprout formation [12], and Jagged1 expressed in breast tumor cells can influence tumor angiogenesis [13]. Similarly, in the context of lymphoma, a specific population of lymphoma cells was shown to up-regulate endothelial Jagged1, through the secretion of FGF4, which in turn up-regulates Notch2 and consequently Hey1 in the tumor cells promoting growth, aggressiveness and resistance to chemotherapy [14]. Finally, a specific Notch1 decoy, that blocks both Jagged ligands interactions with Notch1, was shown to decrease xenograft growth by an anti-angiogenic effect and by the ability to destabilize pericyte-ECs interactions [15].

Therefore, the direct role of endothelial Jagged1 in tumor angiogenesis has not yet been thoroughly described. With this purpose, we have fully characterized tumor growth and progression, and the associated vascular phenotype and cellular metabolic consequences in endothelial *Jag1* mutants in two different mouse tumor models: subcutaneous Lewis Lung Carcinoma (LLC) tumor transplants and in the autochthonous transgenic adenocarcinoma of the mouse prostate (TRAMP) [16, 17].

Here we demonstrate for the first time the effect of directly modulating endothelial Jagged1 in tumor angiogenesis and growth, confirming that loss of endothelial *Jag1* has a strong anti-angiogenic effect that inhibits tumor growth and the acquisition of an invasive phenotype. Moreover, we have shown that endothelial Jagged1 regulates prostatic tumor cell proliferation and de-differentiation by activating Notch3 and consequently up-regulating Hey1 in tumor cells. The results obtained clearly raise the possibility of applying anti-Jagged1 therapies to cancer treatment.

## RESULTS

### Modulation of endothelial *Jag1* interferes with the growth of LLC subcutaneous tumor transplants

To evaluate the contribution of endothelial Jagged1 to tumor angiogenesis, LLC cells were subcutaneously implanted in the dorsum of endothelial specific *Jag1* gain- (e*Jag1*OE) and loss-of-function mouse mutants (e*Jag1*cKO). Tumor volumes (mm^3^) were measured from day seven after the subcutaneous injection until day fourteen.

**Figure 1:**
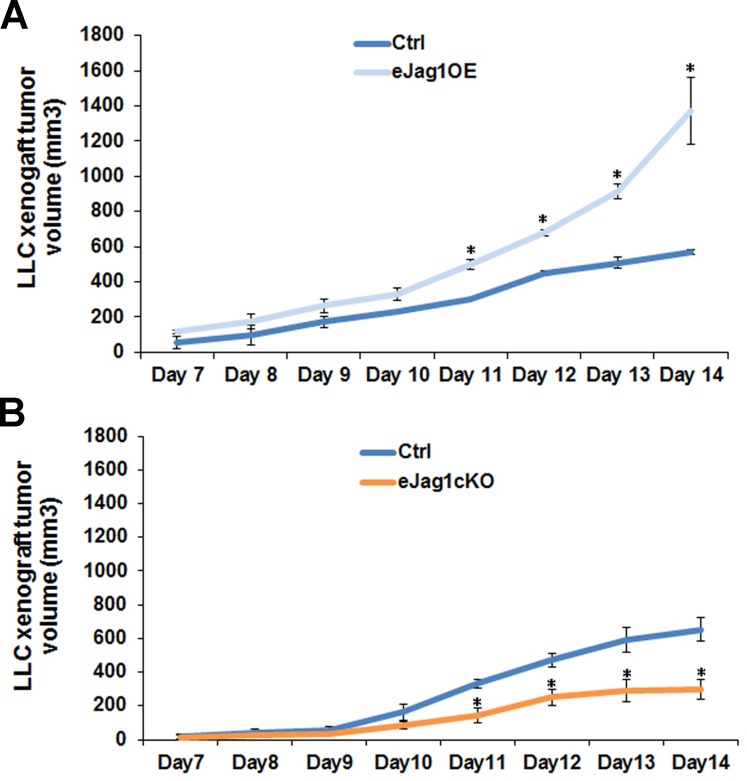
LLC transplant tumor volume in endothelial specific *Jag1* mutants **A.** Progression of LLC transplant tumor volume, from day 7 of subcutaneous injection, in endothelial specific *Jag1* over-expression mutants (Tet-O-Jag Tie2-rtTA^+^) relative to the respective controls (Tet-O-Jag Tie2-rtTA^−^). *Jag1* over-expression mutants present an accelerated growing rate of subcutaneous tumors, with a final tumor volume of more than double of the respective controls. **B.** Progression of LLC transplant tumor volume, from day 7 of subcutaneous injection, in endothelial specific Jag1 Knock-out mutants (*Jag1^lox/lox^* Cre+) and controls (*Jag1^lox/lox^* Cre-). Loss of *Jag1* led to a decrease in the growing rate of subcutaneous tumors, with a final tumor volume of less than half of the respective controls. Results are representative of 2 independent experiments, each with *n* = 6 mice per group. Error bars represent SEM; * represents *p* < 0.05; ** represents *p* < 0.01; *** represents *p* < 0.001.

Endothelial specific *Jag1* overexpression led to significantly accelerated growth of subcutaneous tumors, from day eleven after injection, with a final tumor volume more than two-fold larger (1370 mm^3^) than that of the respective controls (570 mm^3^) (Figure 1A). In contrast, loss of endothelial *Jag1* led to significantly delayed tumor growth, from day eleven after injection (Figure 1B). The average final tumor volume in the endothelial *Jag1* loss-of-function mutants was only 300 mm^3^, less than half of that of the respective controls (650 mm^3^).

### Endothelial Jagged1 contributes to prostate cancer development and progression

After verifying that modulation of endothelial *Jag1* caused such significant alterations in the growth of LLC subcutaneous tumor transplants, we investigated its effect in an autochthonous tumor model. For this end, we crossed the endothelial *Jag1* mutants to a mouse model of prostate adenocarcinoma (TRAMP) [16], which spontaneously develop prostatic lesions from 8 weeks of age [18]. The TRAMP endothelial specific *Jag1* mutants, TRAMP.e*Jag1*OE and TRAMP.e*Jag1*cKO, were sacrificed at 18 and 24 weeks of age, for early and late stages of prostate cancer development, and the prostates collected for analysis.

**Figure 2:**
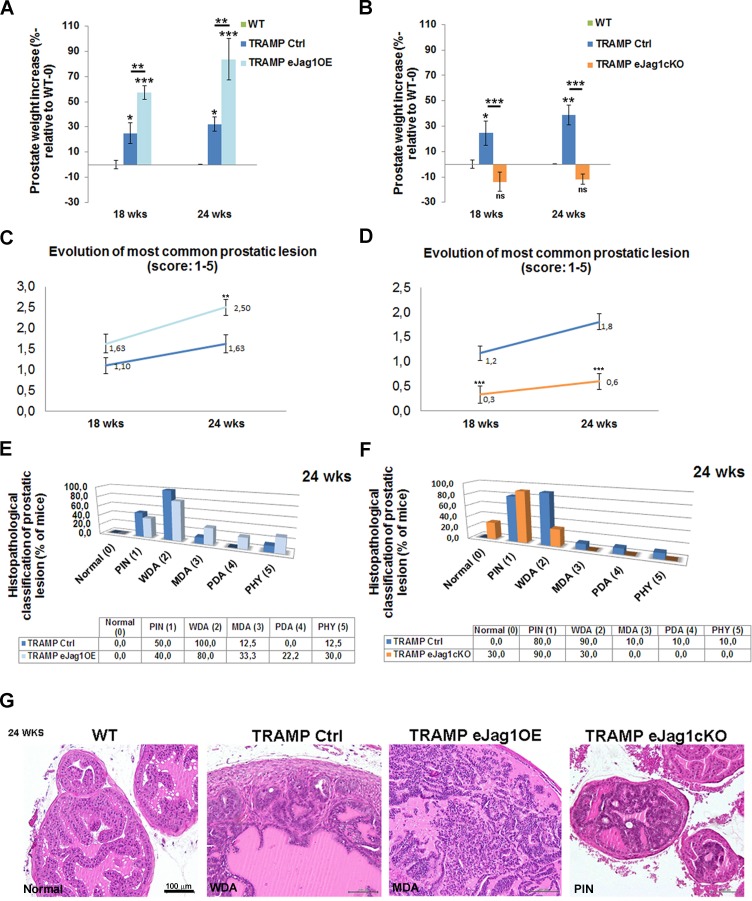
Modulation of endothelial *Jag1* in TRAMP mice **A.** Prostate weight increase (in %, relative to WT-0) in TRAMP Ctrl (Tet-O-Jag Tie2-rtTA^−^) and TRAMP.e*Jag1*OE (Tet-O-Jag Tie2-rtTA^+^) mutants, in early (18 wks) and late (24 wks) stages of prostate tumor development. In both stages TRAMP.e*Jag1*OE present higher prostate weight increase either relative to WT, as to TRAMP Ctrl mice groups. **B.** Prostate weight increase (in %, relative to WT-0) in TRAMP Ctrl (*Jag1^lox/lox^* Cre-) and TRAMP.e*Jag1*cKO (*Jag1^lox/lox^* Cre+) mutants, in early (18 wks) and late (24 wks) stages of prostate tumor development. In both stages TRAMP.e*Jag1*cKO present lower prostate weight increase than TRAMP Ctrl mice group. **C.** and **D.** Evolution of most common prostatic lesion of TRAMP.e*Jag1*OE and TRAMP.e*Jag1*cKO mutants, respectively, and controls, based on histopathological classification of prostatic lesions according to the following score (1-5): Normal (0); prostatic intraepithelial neoplasia (PIN (1)); well differentiated adenocarcinoma (WDA (2)); moderately differentiated adenocarcinoma (MDA (3)); poorly differentiated adenocarcinoma (PD (4)); or phylloides-like cancer (PHY (5)). TRAMP.e*Jag1*OE present a higher score evolution than controls, whereas TRAMP.e*Jag1*cKO present a lower one. **E.** and **F.** Frequency distribution (% of mice) of histophatological classification of prostatic lesions at 24 weeks of age in TRAMP.e*Jag1*OE and TRAMP.e*Jag1*cKO, respectively, versus controls. **G.** H & E representative images of the histopathological classification in WT (no lesions), TRAMP Ctrl (WDA), TRAMP.e*Jag1*OE (MDA) and TRAMP.e*Jag1*cKO (PIN) mice. Results are representative of *n* = 12 per mice group for each time point. Error bars represent SEM; * represents *p* < 0.05; ** represents *p* < 0.01; *** represents *p* < 0.001.

Endothelial specific *Jag1* over-expression TRAMP mice presented increased prostate weights relative to the respective controls (TRAMP Ctrl) at both early and late stages of prostate tumor development (Figure 2A). Accordingly, loss of endothelial *Jag1* caused decreased total prostate weights due to reduction of the tumors, relative to TRAMP Ctrl mice, both in early and late stages (Figure 2B). Noticeably, the prostate weights of TRAMP.e*Jag1*cKO did not differ significantly from those of WT animals, indicating a most considerable reduction in tumor growth.

Histopathological analysis was carried out blindly and the tumors scored according to the following categories: Normal (0), prostatic intraepithelial neoplasia [PIN (1)], well differentiated adenocarcinoma [WDA (2)], moderately differentiated adenocarcinoma [MDA (3)], poorly differentiated adenocarcinoma [PD (4)], or phylloides-like cancer [PHY (5)] [18]. The prostatic lesions evolve in a progressive manner, with different lobes of the prostate presenting different stages of tumor development. Endothelial overexpression of *Jag1* caused an overall acceleration of prostate cancer progression (Figure 2C, 2D and 2F; Suppl. Figure 1A). At an early stage, even though there was no statistically significant difference in the most common lesion score between TRAMP.e*Jag1*OE and the respective controls (Figure 2C), it was observed that, in the controls, the majority of animals (70%) presented lesions of PIN (Suppl. Figure 1A), while in the e*Jag1*OE group, the majority (85,7%) already had evolved to lesions of WDA. Similarly, at a late stage, it was observed a statistically significant difference in the most common lesion score (Figure 2C) between the mouse groups: 100% of control mice (TRAMP Ctrl) presented lesions of WDA and few animals progressed to advanced stages of prostatic adenocarcinoma (Figure 2C and 2D), while the TRAMP.e*Jag1*OE group presented a greater percentage of animals that progressed to advanced stages of prostatic adenocarcinomas (Figure 2C) (33% lesions of MDA, 22% of PDA and 30% PHY lesions).

In contrast, TRAMP.e*Jag1*cKO mutant mice presented a statistically significant inhibition of prostate tumor progression (Figure 2C, 2E and 2G; Suppl. Figure 1B). At an early stage of tumor development (18wks), the respective control group presented a mean score of the most common lesion of 1.2 (Figure 2D) with 66,7% of animals revealing lesions of WDA (Suppl. Figure 1B), while the e*Jag1*cKO mouse group presented a mean score of 0.3 (Figure 2D) with 44,4% animals still showing no lesions (Normal), and the majority (77,8%) progressing only to lesions of PIN (Suppl. Figure 1B). At a later stage, the same kind of response was observed with WDA in 90% of control animals, while only 30% of e*Jag1*cKO evolved to WDA, being the majority (90%) of this latest group classified mainly with lesions of PIN (Figure 2F and 2G).

From the analysis of the most common lesion per animal it was also clear that there was no statistical interaction between the genotype and respective control groups throughout the evolution of the lesions (Figure 2C and 2D), in either TRAMP.*eJag1*cKO or TRAMP.e*Jag1*OE. This means that the effect of modulating endothelial *Jag1* remained constant in time (evolution of tumor progression).

To gain additional confirmation of the differences in the progression and severity of prostatic lesions we immunostained the prostate samples for PSMA, a known marker of prostate cancer progression [19]. Consistently with the prostate weight and histopathological classification data, TRAMP.e*Jag1*OE presented very strong immunostaining for PSMA in the prostatic tissue, while TRAMP.e*Jag1*cKO presented very weak signal, compared with controls (TRAMP Ctrl) (Suppl. Figure 1C).

### Endothelial Jagged1 has a pro-angiogenic function in tumor development

**Figure 3:**
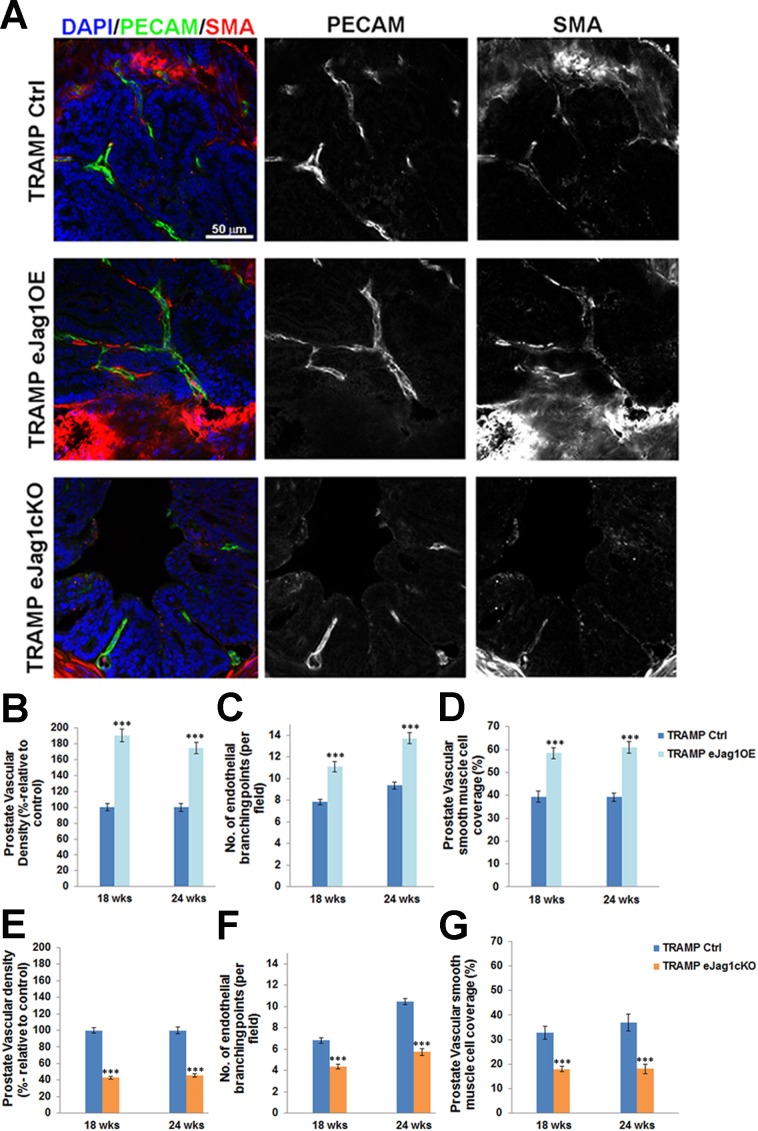
Prostate tumor vascular phenotype in TRAMP endothelial-specific *Jag1* mutants **A.** Representative confocal (one z layer) immunostaining images (40x amplification) marked for PECAM-1 (green) and SMA (red), to evaluate vascular density and vSMC of prostate samples. **B.** Percentage of vascular density (relative to control = 100%) is increased in TRAMP endothelial *Jag1* over-expression mutants as shown by PECAM-1 labeling. **C.** Number of endothelial branching points, demonstrating increased branching in TRAMP.e*Jag1*OE relative to controls. **D.** Percentage of vascular smooth muscle coverage, showing increased levels of SMA on TRAMP *eJag1OE* mutant vasculature, relative to controls. **E.** Percentage of vascular density (relative to control=100%) is decreased in TRAMP endothelial *Jag1* knock-out mutants. **F.** Number of endothelial branching points, demonstrating decreased branching in TRAMP.e*Jag1*cKO relative to controls. **G.** Percentage of vascular smooth muscle coverage, showing decreased levels of SMA on TRAMP *eJag1c*KO mutant vasculature, relative to controls. DAPI (blue) stains nuclei. Error bars represent SEM; * represents *p* < 0.05; ** represents *p* < 0.01; *** represents *p* < 0.001.

Efficient modulation of endothelial Jagged1 was achieved in our conditional gain-of-function and knock-out mutants as demonstrated by the increased and decreased *Jag1* transcription levels in ECs and the increased and decreased fluorescence levels of Jagged1 co-localized with PECAM-1, respectively (Suppl. Figure 2). To address whether the altered growth of subcutaneous LLC tumor transplants and prostate cancer development and progression observed in these EC-specific mutants was indeed associated with altered vessel growth, the vascular morphology of the tumors was examined. The endothelium was visualized by immunostaining against PECAM-1, while α-SMA was used to reveal perivascular cell coverage and thereby analyze vessel maturation (Figures 3A-3G and Suppl. Figure 3).

In e*Jag1*OE mutants, tumor vasculature was denser in both LLC tumor transplants (Suppl. Figure 3A and 3B) and prostate tumors (Figure 3A and 3B), with increased number of endothelial branching points (Figure 3A and 3C). Regarding perivascular coverage, it was observed, despite the abundant SMA positive signal from the stroma surrounding each prostatic gland, that e*Jag1*OE tumor vasculature presented considerably more smooth muscle cells attached to the endothelial wall than the respective controls (Figure 3A and 3D; Suppl. Figure 3A and 3C). Not surprisingly, tumor vasculature of e*Jag1*cKO mutants was the opposite of what was observed in the gain-of-function mutants: sparser (Suppl. Figure 3A and 3D; Figure 3A and 3E), with reduced ramification (Figure 3F), and decreased number of perivascular SMA positive cells (Suppl. Figure 3A and 3E; Figure 3A and 3G). From Figure 3 it is also clear that there are no major differences in tumor vasculature between early (18 wks) and late (24 wks) stages of prostate tumor development.

Tumor endothelial pericyte coverage was also investigated by immunostaining for pdgfr-β and ng-2 [20]. Interestingly, no significant differences were observed in pericyte coverage with any of the markers, in either TRAMP.e*Jag1*OE or TRAMP.e*Jag1*cKO relative to the respective controls (Suppl. Figure 4A-4C). In contrast, in the LLCs transplant model, we observed significantly increased and decreased levels of endothelial pdgfr-β coverage in OE mutants and KO mutants, respectively (Suppl. Figure 5).

**Figure 4:**
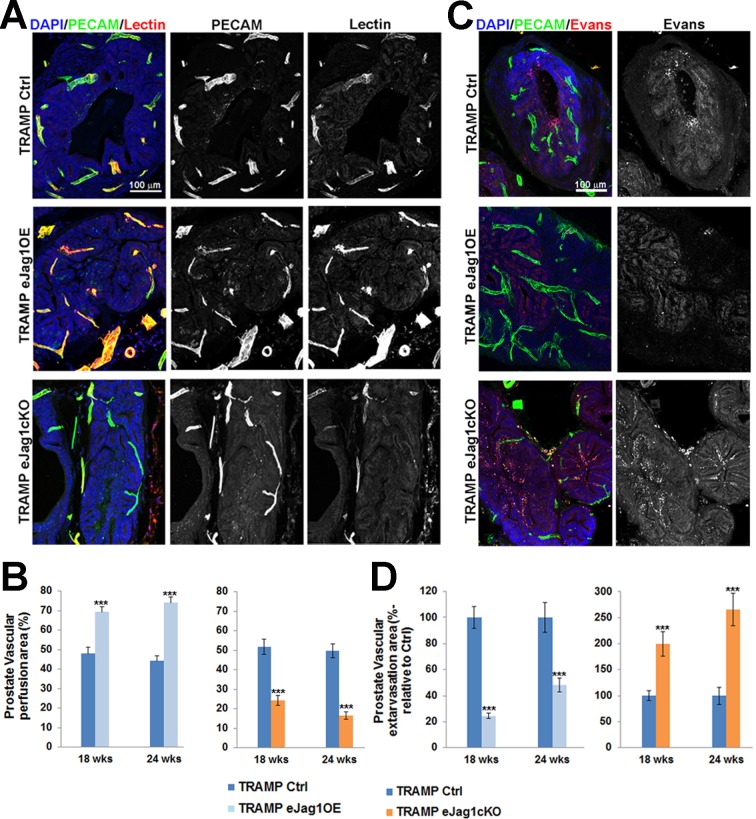
Prostate tumor vascular perfusion and extravasation in TRAMP endothelial-specific *Jag1* mutants **A.** Lectin (red) and PECAM-1 (green) confocal immunostaining (20x amplification) (maximum intensity projections) of TRAMP.e*Jag1*OE and TRAMP.e*Jag1*cKO mutants, to evaluate the co-localization of both signals, indicative of vessel perfusion. **B.** Percentage of perfused area in the total vascular area (given by vascular density measurements) showing increased and decreased lectin labeling in the endothelial *Jag1* over-expression and loss-of-function vasculature, respectively. **C.** Evans’ Blue (red) and PECAM-1 (green) confocal immunostaining (20x amplification) images (maximum intensity projections) showing the extravasation areas. **D.** Percentage of vascular extravasation area in the total vascular area, showing decreased Evans’ Blue staining in TRAMP.*eJag1OE*, and increased in TRAMP.e*Jag1*cKO mutants. DAPI (blue) stains nuclei. Error bars represent SEM; *** represents *p* < 0.001.

Tumor vessel functionality in terms of perfusion and leakage was also analyzed by biotinylated lectin perfusion and Evans’ Blue dye, respectively (Figure 4 and Suppl. Figure 6). Over-expression of *Jag1* in the endothelium was associated with an increased number of perfused, lectin-containing vessels, whereas endothelial *Jag1* loss-of-function led to a significant decrease in vessel perfusion relative to the respective controls. Moreover, Evans’ Blue extravasation was significantly reduced in e*Jag1*OE mutants while e*Jag1*cKO mutants presented an increased vascular extravasation area. These differences in tumor vascular phenotypes were observed in both tumor models used, prostatic tumors [early (18wks) and late (24 wks) stages, Figure 4] as well as LLC subcutaneous tumor transplants (Suppl. Figure 6).

Taken together, endothelial *Jag1* over-expression led to the formation of a dense, mature, and more functional tumor vascular plexus, that contributes to increased tumor growth and progression. Conversely, endothelial *Jag1* loss-of-function led to a sparse, immature, and poorly functional neo-vessel network that substantially inhibits tumor growth.

### Endothelial Jagged1 elicits changes in the transcription profile of angiocrine factors of endothelial and perivascular tumor associated cells

**Figure 5:**
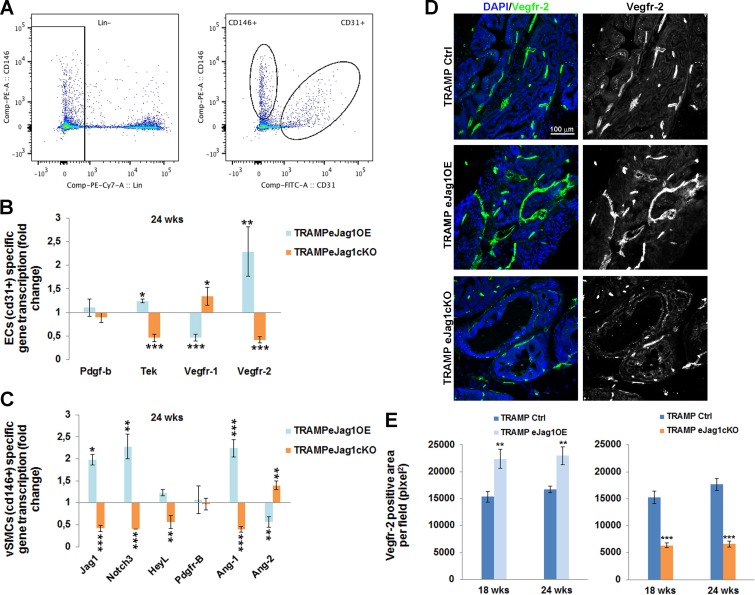
Transcription profile of angiocrine factors by endothelial and perivascular tumor associated cells in TRAMP endothelial-specific *Jag1* mutants RNA was isolated from prostates collected at the end-point, and gene transcript analysis was performed by quantitative real-time RT-PCR for genes involved in angiogenesis. **A.** ECs (Lin^−^ (cd45^−^ ter119^−^) cd31^+^) and vSMCs (Lin^−^ (cd45^−^ ter119^−^) cd146^+^cd31^−^) sorted populations for specific gene transcription analysis. **B.** ECs specific relative gene transcription. **C.** vSMCs specific relative gene transcription. Gene transcript levels were normalized to PECAM-1 mRNA levels, and the house-keeping gene β-actin was used as endogenous control. Blue bars represent the gene expression levels of samples collected from *eJag1*OE mutants, and orange bars the gene expression levels from *eJag1cK*O mutants, relative to the respective controls. **D.** Representative images of Vegfr-2 immunofluorescence (green) (20x amplification) in TRAMP endothelial-specific *Jag1* mutants. **E.** Quantification of Vegfr-2 positive area per field (pixel^2^) demonstrating increased stained areas in TRAMP.e*Jag1*OE and decreased staining in TRAMP.e*Jag1*cKO relative to respective controls. DAPI (blue) stains nuclei. Error bars represent SEM; * represents *p* < 0.05; ** represents *p* < 0.01; *** represents *p* < 0.001.

In order to better understand the molecular mechanisms behind the tumor vascular phenotypes observed in *eJag1*OE and *eJag1*cKO mutants, we performed RT-qPCR analysis of selected genes (Figure 5). RNA was extracted from ECs (Lin^−^ (ter119^−^cd45^−^) cd31^+^) and vSMC cells (Lin^−^ (ter119^−^cd45^−^) cd146^+^cd31^−^) FACS sorted from prostate samples collected at early and late stages of tumor development (Figure 5A).

ECs specific gene transcription (Figure 5B and Suppl. Figure 7A), revealed that the levels for *Pdgfb* transcription, encoding PDGF-B, the endothelial ligand for PDGFRβ, which controls the recruitment of pericytes, was not significantly altered in either of the mutants (Figure 5B), even though a significant down-regulation in *eJag1cKO* early stage prostate samples was observed (Suppl. Figure 7A). *Tek* (encoding the Tie2 receptor tyrosine kinase) which regulates vascular permeability and maturation [21] was downregulated in *eJag*1cKO mutants and increased in gain-of-function mutants at both time points. Regarding vascular endothelial growth factor receptor-1 (*Vegfr1/Flt1*) transcription in prostate tumor samples we observed a down-regulation in TRAMP.e*Jag1*OE and an up-regulation in TRAMP.e*Jag1*cKO. In contrast, *Vegfr-2* (*Vegfr2/Kdr/Flk1*) levels were positively modulated by endothelial Jagged1, with up-regulation and down-regulation in OE and KO prostate samples, respectively.

Furthermore, mural cell specific transcription analysis (Figure 5C and Suppl. Figure 7B) revealed a downregulation of *Jag1, Notch3* and *HeyL* (perivascular cell Notch effector) in e*Jag1*cKO and an upregulation in e*Jag1*OE mutants prostates. Additionaly, *PdgfrB* (encoding PDGFRβ) levels were not altered in response to endothelial Jagged1 modulation, as already demonstrated by protein staining for the receptor. *Ang1* (perivascular ligand for Tie2 receptor), was up-regulated in OE and down-regulated in KO mutants, On the contrary, *Ang2* (antagonistic ligand for Tie2 receptor) was down-regulated in OE and up-regulated in KO mutants,

These results indicate that *Jag1* modulation in the endothelium is able to ellicit changes in the expression profiles of angiocrine factors that regulate angiogenesis and the recruitment of mural cells.

### Modulation of neo-vasculature of prostatic tumors leads to alterations in local hypoxic levels

**Figure 6:**
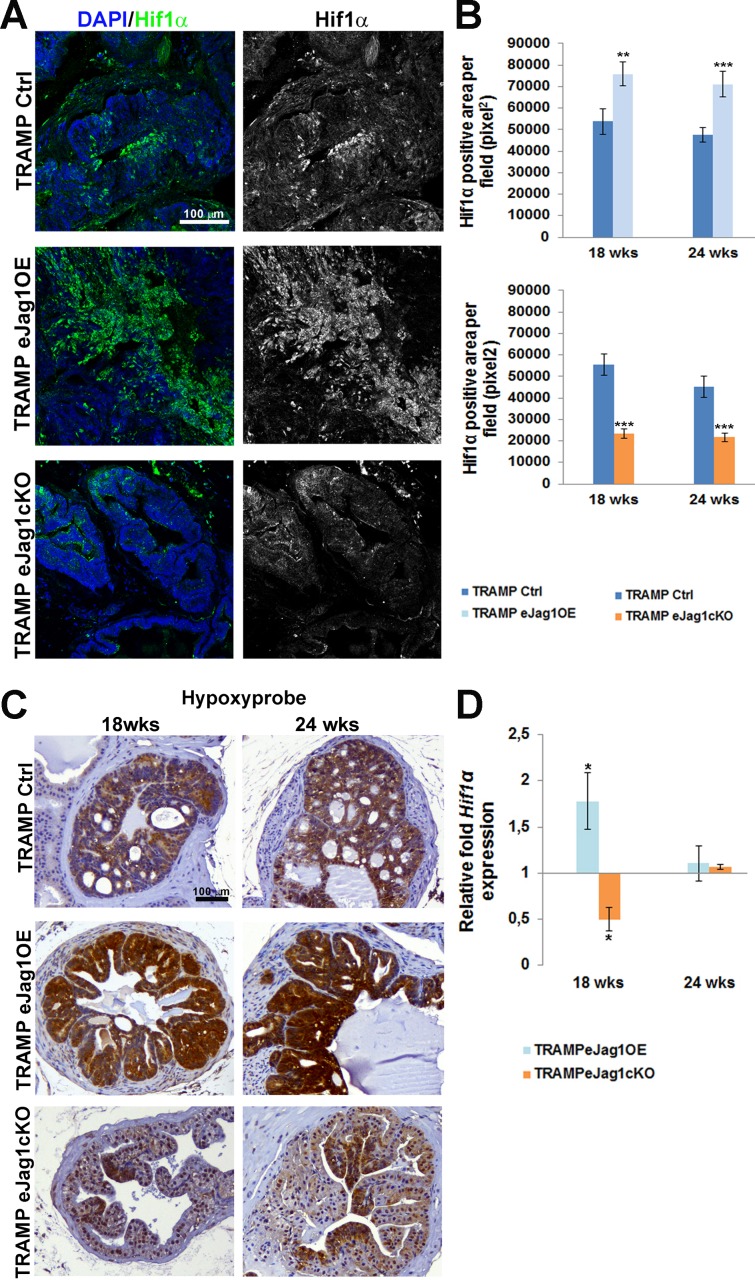
Prostate tumor hypoxic levels in TRAMP endothelial-specific *Jag1* mutants **A.** Representative images of Hif1α immunofluorescence (green) (20x amplification) (maximum intensity projections) in TRAMP endothelial-specific *Jag1* mutants (OE and KO). **B.** Quantification of Hif1α positive area per field (pixel^2^) demonstrating increased stained areas in TRAMP.e*Jag1*OE and decreased staining in TRAMP.e*Jag1*cKO relative to respective controls. **C.** Hypoxypyprobe immunohistochemical staining, in early (18 wks) and late (24 wks) stages of tumor progression, showing strong positive staining in TRAMP.e*Jag1*OE while TRAMP.e*Jag*1cKO mutants present weak positive signal, compared with controls (TRAMP Ctrl). **D.** Relative fold *Hif1α* mRNA expression showing increased expression in TRAMP.e*Jag1*OE and decreased expression in TRAMP.e*Jag1*cKO mutants, at 18 weeks of age. DAPI (blue) stains nuclei. Error bars represent SEM; * represents *p* < 0.05; ** represents *p* < 0.01; *** represents *p* < 0.001.

After characterizing the neo-vasculature of two different tumor models in endothelial *Jag1* specific mutants, we aimed to understand how the different vascular phenotypes were able to cause such significant differences in progression of prostatic cancer in mice. Therefore, in order to evaluate tumor hypoxia, immunostaining for Hif1α was performed in prostatic samples from TRAMP e*Jag1* mutants. As can be observed in Figure 6A and 6B, e*Jag1*OE mutants presented increased levels of Hif1α, whereas e*Jag1*cKO show decreased levels, relative to the respective controls, in either early (18 wks) or late (24 wks) stages of tumor progression.

In addition, hypoxyprobe was administered to mice prior to dissection in order to visualize the tumor areas with low oxygen pressure (pO2 = 10 mmHg) (Figure 6C). The response observed was consistent with Hif1α staining in both TRAMP.e*Jag1*OE, with stronger and extended areas of positive staining, and TRAMP.e*Jag1*cKO mutants, that presented only weak and localized staining. Transcript levels of *Hif1α* mRNA were also analyzed by qRT-PCR (Figure 6D). *Hif1α* mRNA levels varied in the same manner as the protein staining, with up-regulation in OE and down-regulation in KO mutants, but only in an early stage (18 wks). Surprisingly, in a late stage (24 wks) no differences were observed between the different mutants and the respective controls.

### Endothelial Jagged1 induces proliferation and inhibits apoptosis in the surrounding tumor tissues

**Figure 7:**
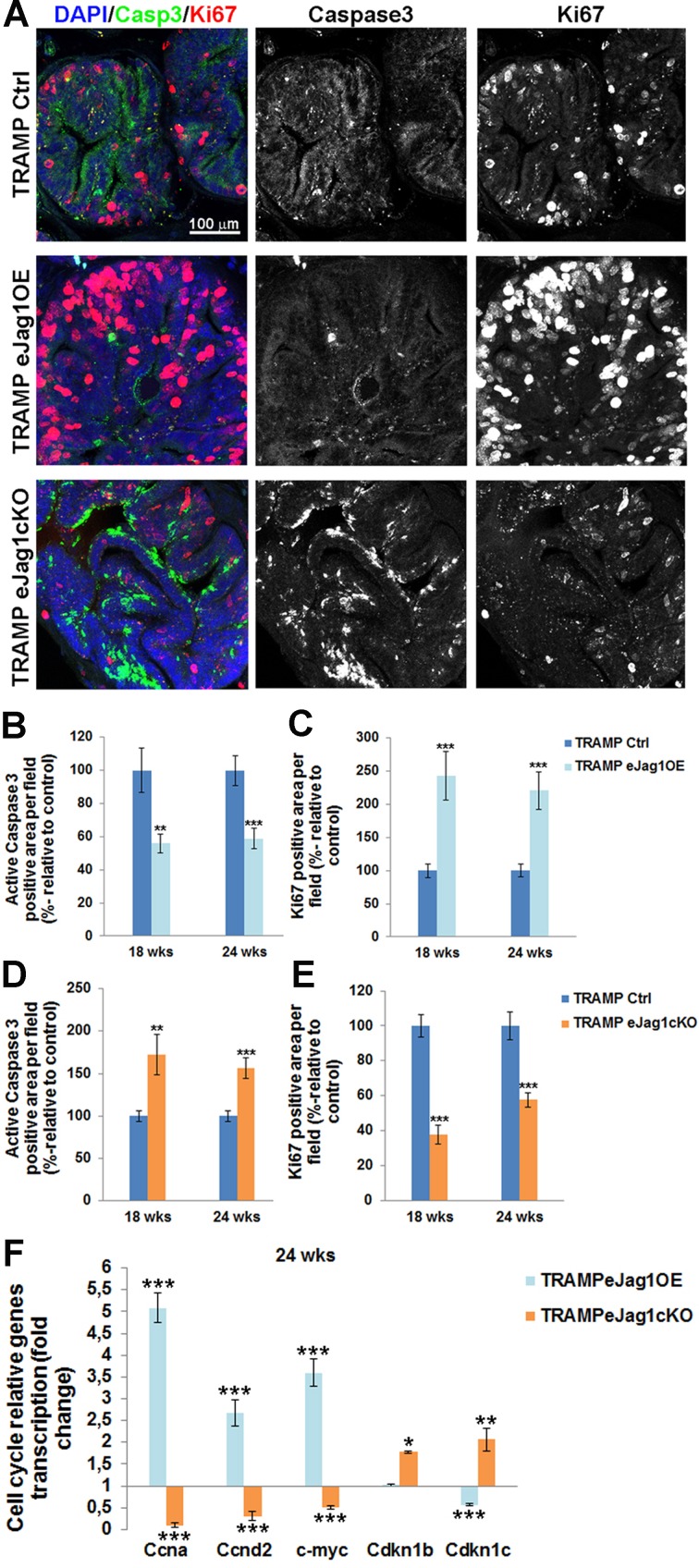
rostate cellular apoptosis and proliferation in TRAMP endothelial-specific *Jag1* mutants **A.** Representative images of active Caspase3 (green) and Ki67 (red) immunofluorescence staining (20x amplification) (maximum intensity projections) in TRAMP endothelial-specific *Jag1* mutants (OE and KO). **B.** Prostatic lesions of TRAMP.e*Jag1*OE mutants presented decreased percentage of active caspase3 positive area per field, relative to control (100%), either at 18 as at 24 weeks of age. **C.** Prostate samples from TRAMP.e*Jag1*OE presented increased percentage of Ki67 positive area per field, relative to control (100%), at both time points (18 and 24 wks) **D.** Prostatic lesions of TRAMP.e*Jag1*cKO mutants presented increased percentage of active caspase3 positive area per field, relative to control (100%), at 18 and 24 weeks of age. **E.** Prostate samples from TRAMP.e*Jag1*cKO presented decreased percentage of Ki67 positive area per field, relative to control (100%), at both time points (18 and 24 wks). **F.** Relative fold mRNA expression of cell cycle regulatory genes in TRAMP.e*Jag1*OE and TRAMP.e*Jag1*cKO mutants, at 24 weeks of age. DAPI (blue) stains nuclei. Error bars represent SEM; * represents *p* < 0.05; ** represents *p* < 0.01; *** represents *p* < 0.001.

To better understand the metabolic changes in prostatic tumor development caused by altered vascular supply, cellular apoptosis and proliferation were addressed by immunostaining for active caspase 3 and ki67, respectively, on prostate samples from TRAMP.e*Jag1* mutants (Figure 7). Endothelial *Jag1* overexpression in TRAMP mice (TRAMP.e*Jag1*OE) led to decreased apoptosis (Figure 7A and 7B) and increased cellular proliferation (Figure 7A and 7C). On the other hand, endothelial *Jag1* loss-of-function (TRAMP.e*Jag1*cKO) resulted in increased cellular apoptosis (Figure 7A and 7D) and decreased proliferation (Figure 7A and 7E).

Moreover, we profiled the transcription of several important cell-cycle regulatory genes in TRAMP.e*Jag1* prostate samples (Figure 7F). There was up-regulation and down-regulation of the cell-cycle stimulating genes, *Ccna* (encoding for Cyclin A), *Ccnd2* (encoding for CyclinD2), and *c-myc,* in OE and KO samples, respectively. Conversely, the opposite response was observed in the cell-cycle inhibitors, *Cdkn1b* (*p27*, encoding for Cyclin-Dependent Kinase Inhibitor 1B) and *Cdkn1c* (*p57*, encoding for Cyclin-Dependent Kinase Inhibitor 1C): in TRAMP.e*Jag1*OE prostate samples *Cdkn1c* was down-regulated while in TRAMP.e*Jag1*cKO both kinase inhibitors were up-regulated.

### Modulation of endothelial *Jag1* leads to alterations in epithelial-to-mesenchymal transition (EMT)

Lastly, we intended to investigate if the alterations in vascular supply of the prostate tumors, and consequently altered metabolism of tumor cells, would contribute to increased and/or decreased pressure for the acquisition of an invasive phenotype and to epithelial-to-mesenchymal transition. To this purpose, we performed immunostaining for the epithelial adhesion marker, E-cadherin, and for Snail, a transcription factor known for the induction of EMT [22], in the prostatic lesions of TRAMP mice.

**Figure 8:**
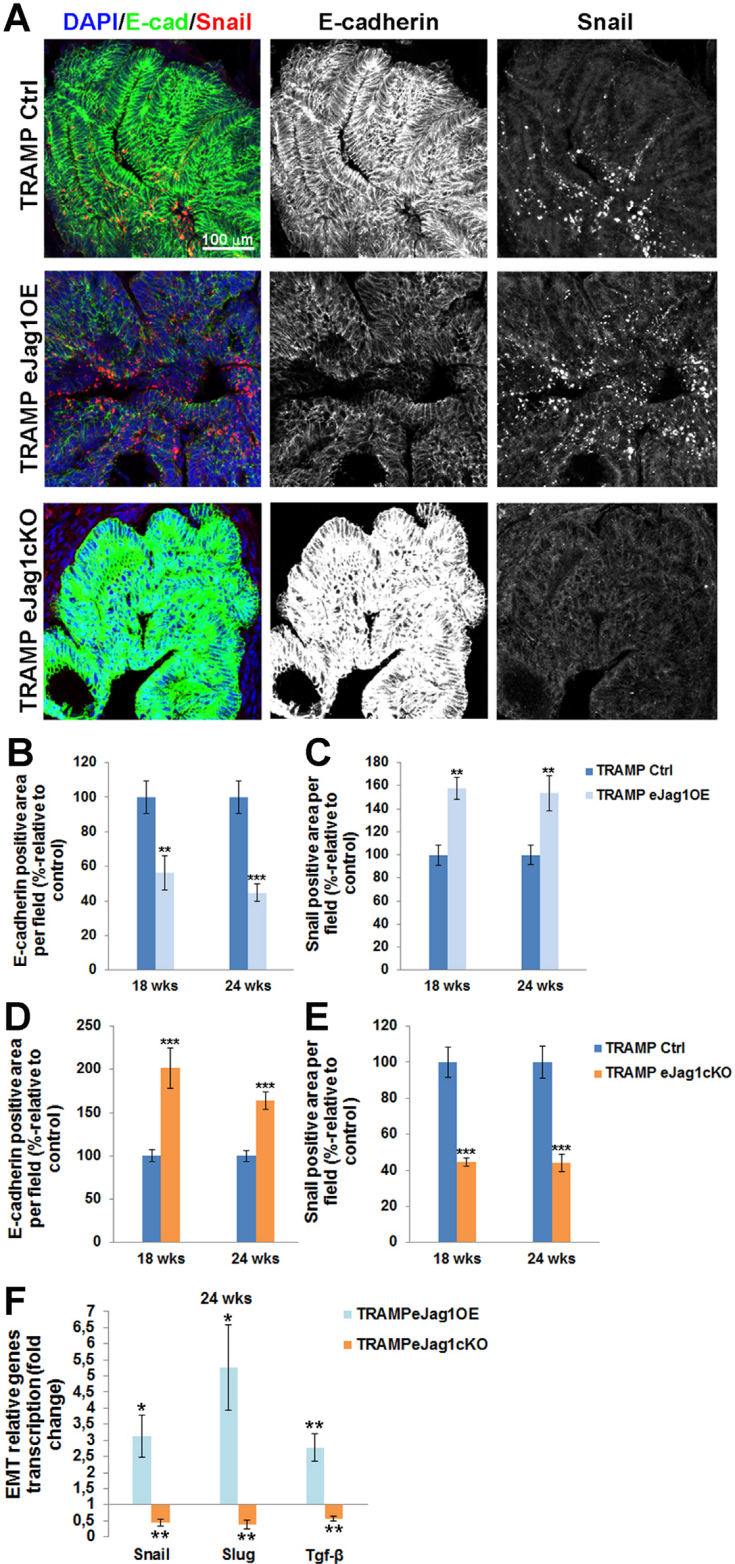
Epithelial-to-mesenchymal transition in prostate lesions of TRAMP endothelial-specific *Jag1* mutants **A.** Representative images of E-cadherin (green) and Snail (red) immunofluorescence staining (20x amplification) (maximum intensity projections) in TRAMP.e*Jag1*OE and TRAMP.e*Jag1*cKO mutants. **B.** Decreased percentage of E-cadherin per field in TRAMP.e*Jag1*OE mutants, relative to respective controls (100%), at 18 and 24 weeks of age. **C.** Increased Snail positive area per field in OE mutants, relative to respective controls (100%). **D.** Increased percentage of E-cadherin per field in TRAMP.e*Jag1*cKO mutants, relative to respective controls (100%), at 18 and 24 weeks of age. **E.** Decreased Snail positive area per field in KO mutants, relative to respective controls (100%). **F.** Relative fold of *Snail, Slug* and *Tgf-β* mRNA expression, demonstrating increased expression in the prostate of TRAMP.e*Jag1*OE mutants whereas in TRAMP.e*Jag1*cKO mutants prostate their expression is decreased at 24 weeks of age. DAPI (blue) stains nuclei. Error bars represent SEM; * represents *p* < 0.05; ** represents *p* < 0.01; *** represents *p* < 0.001.

Over-expression of endothelial *Jag1* (TRAMP.e*Jag1*OE) was associated with substantial loss of E-cadherin expression (Figure 8A and 8B) and increased expression of Snail (Figure 8A and 8C) relative to the respective controls. Conversely, both *Snail* and *Slug* mRNA expression levels were increased in these mutants (Figure 8F). In contrast, loss of endothelial *Jag1* (TRAMP.e*Jag1*cKO) was associated with increased E-cadherin expression (Figure 7A and 7D) and decreased Snail expression (Figure 8A and 8E), relative to the respective controls. In these mutants the levels of mRNA expression of both *Snail* and *Slug* were also decreased (Figure 8F). We also analyzed *tgf-β* transcription levels, since it is a known Jagged1 dependent regulator of EMT [23], and observed increased and decreased transcription in OE and KO prostates, respectively (Figure 8F).

### Endothelial Jagged1 exerts its angiogenic function through Notch4/Hey1 and its angiocrine function through Notch3/Hey1 influencing tumor cell proliferation and de-differentiation

Having previously established that endothelial Jagged1 is able to activate Notch4 in a physiological angiogenic response [11], we wanted to confirm this in a tumor setting. To do it, we immunostained the intracellular domain of Notch4 (N4ICD) in our TRAMP.e*Jag1* mutants and co-localized it with PECAM to evaluate endothelial activation of Notch4 (Suppl. Figure 8). In TRAMP.e*Jag1*OE prostates we observed increased double positive staining for N4ICD and Pecam (Suppl. Figure 8A and 8B) whereas in TRAMP.e*Jag1*cKO prostates N4ICD staining was decreased in the endothelium (Suppl. Figure 8A and 8C).

**Figure 9:**
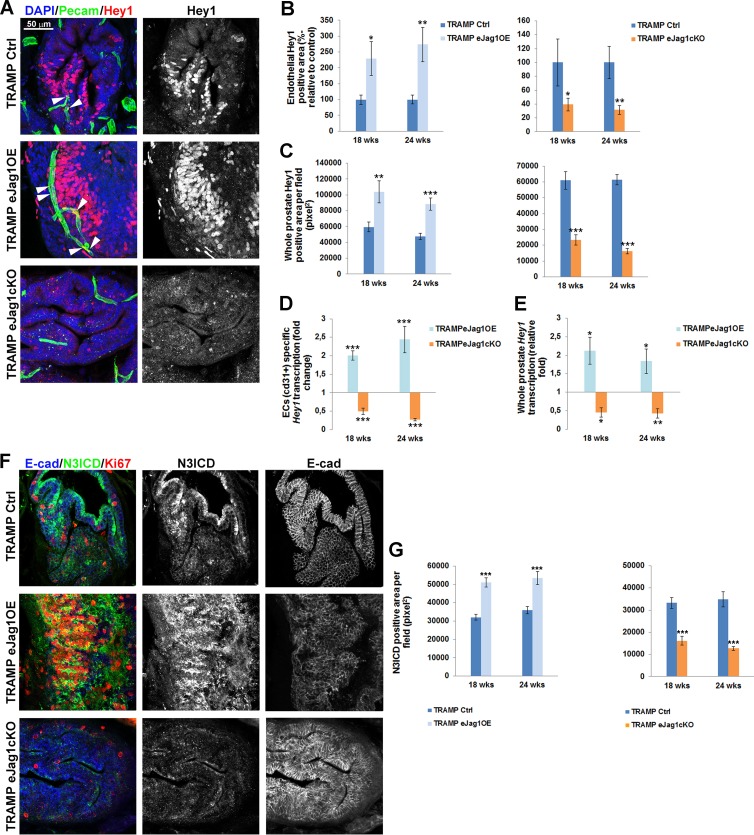
Hey1 transcription and expression and Notch3 intracellular domain (N3ICD) expression in prostate lesions of TRAMP endothelial-specific *Jag1* mutants **A.** Representative images of Hey1 (red) and Pecam (green) immunofluorescence staining (40x amplification) (maximum intensity projections) in TRAMP.e*Jag1*OE and TRAMP.e*Jag1*cKO mutants. White arrows indicate Hey1 expression in endothelial cells. **B.** Quantification of endothelial Hey1 positive area per field (%-relative to control) in TRAMP.e*Jag1*OE (left) and TRAMP.e*Jag1*cKO (right), demonstrating increased and decreased double positive staining for Hey1/Pecam in e*Jag1* mutants, respectively. **C.** Quantification of whole prostate Hey1 positive area per field (pixel^2^), demonstrating increased and decreased areas in OE (left) and KO (right) mutants, respectively. **D.** and **E.** ECs specific and whole prostate Hey1 transcription analysis in TRAMP.e*Jag1* prostates. **F.** Representative images of N3ICD (green), E-cadherin (blue) and Ki-67 (red) immunofluorescence staining (40x amplification) (maximum intensity projections) in TRAMP.e*Jag1*OE and TRAMP.e*Jag1*cKO mutants. **G.** Quantification of N3ICD positive area per field (pixel^2^), demonstrating increased and decreased areas, relative to controls, of e*Jag1*OE and e*Jag1*cKO prostates, respectively. DAPI (blue) stains nuclei. Error bars represent SEM; * represents *p* < 0.05; ** represents *p* < 0.01; *** represents *p* < 0.001.

In our previous study [11], Hey1 was the main Notch effector found downstream of Jagged1/Notch4 signaling. Therefore we aimed to quantify Hey1 in the vasculature of e*Jag1* mutants. As shown in Figure 9, in TRAMP.e*Jag1*OE mutants prostates there were increased levels of Hey1 staining in the endothelium, while in TRAMP.e*Jag1*cKO prostates they were decreased (Figure 9A and 9B). Interestingly, we observed increased and decreased Hey1 staining in tumor cells adjacent to the vessels (Figure 9A and 9C), in OE and KO samples, respectively, relative to controls. Additionally, Hey1 modulation by endothelial Jagged1 was confirmed at the transcript level (Figure 9D and 9E) in both ECs specific and whole prostate mRNA analysis. TRAMP.e*Jag1*OE mutants presented *Hey1* up-regulation in ECs (Figure 9D) and in whole prostate (Figure 9E), whereas e*Jag1*cKO mutants presented a down-regulation response.

Given the significant activation of Notch signaling, by Hey1 transcription and expression, observed in tumor cells adjacent to the vasculature, we hypothesized that a specific Notch receptor was being activated by endothelial Jagged1. Endothelial Jagged1 has been shown to be able to activate Notch3 in adjacent perivascular cells [24]. Additionally, high levels of Notch3 have been described in prostate cancer cells with high metastatic potential [25]. Therefore, we hypothesized that endothelial Jagged1 could also be acting as an angiocrine factor activating Notch3 in adjacent tumor cells, and consequently regulating proliferation and de-differentiation. To test this hypothesis, we immunostained TRAMP.e*Jag1* samples for N3ICD concomitantly with Ki67 and E-cadherin (Figure 9F and 9G). TRAMP.e*Jag1*OE mutants presented increased staining for N3ICD whereas e*Jag1*cKO mutants presented decreased staining, relative to controls. Remarkably, as shown in Figure 9F, the tumor areas that display activated Notch3 staining also have increased ki67 positive staining and loss of E-cadherin.

## DISCUSSION

In the last decades tumor angiogenesis has become a very active area of research, resulting in the introduction of anti-angiogenic drugs in cancer therapy, such as the anti-VEGF antibody bevacizumab [26] and the tyrosine kinase inhibitors like sunitinib or sorafenib [27]. Many other molecules have been investigated since for their effect on angiogenesis. Modulation of endothelial *Jag1* was previously shown to be crucial in developing retina vascularization [10]. The results presented here describe the effect of modulating endothelial *Jag1* in tumor angiogenesis and metabolism and consequently in tumor development and progression.

We observed that endothelial *Jag1* over-expression accelerated the growing rate of LLC subcutaneous tumor transplants and contributed to the progression and development of prostate cancer in TRAMP mice. This effect was associated with an increase in the density and branching of the tumor vessels. In contrast, endothelial *Jag1* loss-of-function delayed the growing rate of LLC subcutaneous transplants and inhibited the development of prostate lesions in TRAMP mice, by decreasing the density and branching of the tumor neo-vasculature. This appears to be consistent with a report that increased tumor microvascular density (MVD) constitutes a bad prognostic indicator in several solid tumors that induce significant angiogenesis [28].

From the observations in the TRAMP model, where there is a stepwise progression of tumor development, no major differences were observed in the vascular response between early (18 wks) and late (24 wks) stages, which is thought to be a consequence of the angiogenic switch occurring relatively early on the onset of prostatic lesions and therefore before 18 wks of age [29]. In this report we show that endothelial Jagged1 acts as a pro-angiogenic ligand in a tumor setting, after having recently demonstrated this effect in a regenerative setting [11], where Jagged1 antagonizes Dll4 regulation of endothelial branching, by its ability to block Dll4/Notch1 activation and thus by positively regulating Vegfr-2 transcription. Here, we have further validated and complemented the mechanistic process by which endothelial Jagged1 exerts its pro-angiogenic function, by showing that it not only positively regulates Vegfr-2 transcription and expression, but that it also negatively regulates Vegfr-1 transcription, specifically in ECs. Accordingly, a recent report using a Notch decoy that specifically blocks Jagged ligands mediated interaction [15] has shown that the anti-angiogenic effect observed is likely due to increased secretion of the soluble form of Vegfr-1, and thus decreased Vegf/Vegfr-2 signaling.

In addition, in confirming the pro-angiogenic function of endothelial Jagged1 in tumors, we have identified a new role for it in promoting blood vessel maturation in tumor angiogenesis, since e*Jag1*OE tumor vasculature presented increased coverage of SMA+ cells, whereas the vasculature of e*Jag1*KO mutants presented the opposite phenotype. Moreover, the ECs and SMCs specific mRNA levels of *angpt1* (encoding angiopoetin1) and *tek* (encoding tie-2 receptor), respectively, members of one of the main signaling pathways involved in the recruitment of support cells to the vessel wall [30], also responded accordingly with modulation of e*Jag1.* Additionally, endothelial Jagged1 was also able to positively regulate vSMC specific *Jag1* and *Notch3 and HeyL* levels, supporting the existing model where activation of perivascular Notch3 and HeyL effector is essential for the assembly of a SM layer [24]. The contribution of endothelial Jagged1 to vSMC recruitment has already been described in other angiogenic settings [24, 31, 32]. Moreover, we have also suggested previously that the perivascular phenotype observed in e*Jag1* mutants can also be a consequence of Notch4 activation by endothelial Jagged1. Inclusively we have not only demonstrated increased and decreased levels of active Notch4 in OE and KO mutants, respectively, but also increased vessel maturation upon administration of a Notch4 specific agonist to WT mice [11]. Here, we have further validated Notch4 as a strong endothelial receptor for Jagged1.

In tumors, pericyte coverage decrease and perturbed associations between pericytes and endothelial layer have been described [33, 34]. However, we found no significant alterations in pericyte number by pdgfr-β or ng-2 immunostaining or changes in the levels of *pdgfr-β* mRNA in e*Jag1* mutant's prostate tumor vasculature, suggesting that the observed changes in vascular maturation are independent of pericyte coverage. Nonetheless, in the LLCs transplant model, modulation of endothelial Jagged1 produced alterations in pericyte coverage, suggesting that the absence of effect on pericyte coverage by Jagged1 function modulation seems to be prostate specific. Similarly, the use of a Jagged specific Notch decoy [15], presented the same perivascular phenotype as the one caused by endothelial Jagged1 loss-of-function.

Modulation of tumor angiogenesis and maturation by endothelial Jagged1 led to alterations in functionality and permeability of tumor vessels, which ultimately may lead to different hypoxic and metabolic responses of tumor cells. Over-expression of endothelial *Jag1* culminated in increased perfusion and decreased extravasation, while endothelial loss-of-function caused the tumor vasculature to be less perfused and leakier. This was likely to cause, respectively, increased and decreased delivery of oxygen to tumor cells. However, unexpectedly, quantification of Hif1α protein and mRNA levels, as well as pimonidazole- thiol adducts formation by hypoxyprobe administration, indicates that OE and KO mutant prostatic tissues have increased and decreased hypoxic levels, respectively. It is known that Hif1α is up-regulated in most prostate tumor tissues, compared with normal and benign prostate tissues [35]. Additionally, it is also established that prostate cancer cells have the ability to compensate the lack of oxygen by anaerobic glycolytic respiration [36], which is able to persist upon neovascularization, suggesting that the glycolytic phenotype arises from genetic or epigenetic changes [37].

It seems clear that from the early beginning of tumor epithelial transformation (demonstrated in lesions of TRAMP.*eJag1cKO*) the local concentration of oxygen drops in the affected areas. It is also clear that the oxygen concentration levels are inversely proportional to the degree of dysplasia. We have observed that endothelial Jagged1 contributes to tumor dysplasia through two distinct effects: a pro-angiogenic effect, increasing tumor vascular density, maturation and perfusion; and an angiocrine effect likely through Notch3/Hey1 stimulation of tumor cell proliferation. Therefore we propose that the angiogenic and angiocrine functions of endothelial Jagged1 both contribute to increase proliferation and reduce apoptosis leading to increased consumption of local oxygen with consequent acidotic microenvironment conditions and increased hypoxia. Therefore the TRAMP.*eJag1OE* prostates, that present the most aggressive lesions, also present the lowest oxygen levels.

Moreover, numerous reports have also described a crosstalk between Notch and hypoxia signaling pathways [38]. Inclusively, the existence of a negative feed-back loop has been suggested, in order to prevent excessive hypoxic gene induction, by the ability of Hey factors to repress Hif1α induced gene expression [39]. This negative feed-back loop may explain why *Hif1α* mRNA levels were only altered in an early stage (18 wks) of prostate tumor development in TRAMP.e*Jag1* mutants since at late stages (24 wks) modulation of *Hey1* mRNA levels may have caused a repressive effect.

The hyper-productive angiogenesis observed in the prostatic lesions of TRAMP.e*Jag1*OE mutants, was associated with increased proliferation and survival of tumor cells and to the acquisition of a more invasive phenotype promoting de-diferentiation and epithelial-mesenchymal transition (Figure 7A-7C). Accordingly, Jagged1 mediated activation is known to induce epithelial-mesenchymal transition [40]. Conversely, the anti-angiogenic phenotype observed in TRAMP.e*Jag1*cKO mutants was associated with reduced proliferation of tumor cells and increased apoptotic events (Figure 6A, 6D-6E) that ultimately restricted invasiveness (Figure 7A, 7D-7E). Notably, endothelial Jagged1 regulated the transcription profile of several cell cycle regulatory genes: CyclinA, D2 and c-myc were positively regulated while the inhibitors of these kinases activity, Cdkn1b and c were negatively regulated by endothelial Jagged1 function. Similarly, previous studies have demonstrated that down-regulation of *Jag1* induces cell growth inhibition and S phase cell cycle arrest in prostate cancer cells [41].

The metabolic changes observed in prostate tumor cells that arise from altered tumor angiogenic response may not only be a consequence of altered support of nutrients and oxygen, but can also be a consequence of paracrine signaling. In this work we have also unveiled a new angiocrine effect of the Jagged1 ligand. Endothelial Jagged1 not only up-regulated ECs specific Hey1 transcription and expression but also increased its expression in adjacent tumor cells. In twin slides, we could also observe that the same Hey1 positive tumor areas concomitantly expressed active Notch3, had increased tumor cell proliferation and presented loss of epithelial markers, suggesting a de-differentiation phenotype. Therefore, we suggest that endothelial Jagged1 is able to regulate tumor cell metabolism by its angiocrine function through Notch3/Hey1. Accordingly, Jagged1 expressing EC- tumor cell signaling has been described in the regulation of colorectal cancer [42], and in the ability of providing chemo resistance, aggressiveness to lymphoma cells by activating Notch2 and consequently Hey1 in these adjacent cells [14].

The angiocrine effect combined with the pro-angiogenic and pro-maturation function of endothelial Jagged1 may constitute an important therapeutic advantage over Dll4 based-therapies. Blockade of DLL4 was shown to lead to increased nonproductive tumor vasculature inhibiting tumor growth [5, 6]. However, long-term blockade of Dll4 was found to lead to the development of vascular neoplasms [43] and other toxicities [44].

In summary, this study is the first to demonstrate the effect of directly modulating endothelial *Jag1* in tumor development. Notably, loss of endothelial *Jag1* not only had an inhibitory effect in the neo-angiogenic and maturation responses but also had an angiocrine effect, through inhibition of Notch3/Hey in tumor cells, restricting proliferation, increasing apoptosis, and preventing the acquisition of an invasive phenotype by tumor cells, therefore inhibiting growth and development of subcutaneous LLC tumor transplants and autochthonous prostatic tumors in mice. Thus, this report provides substantial support for the development of novel therapeutic strategies against cancer based on blocking endothelial Jagged1 function.

## MATERIALS AND METHODS

### Experimental animals

All the procedures involving animals used in this study were approved by the Ethics and Animal Welfare Committee of the Faculty of Veterinary Medicine of Lisbon (see Suppl. Methods).

To obtain the gain-of-function mutants, heterozygous Tet-O-Jag mice were crossed with a line of heterozygous Tie-2-rtTA mutant mice. The double heterozygous offspring obtained, Tet-O-Jag; Tie-2-rtTA, were administered doxycicline (4mg/ml in drinking water from week 4), in order to activate the overexpression of *Jag1* under the control of the *Tie-2* promotor. One control group contained mice with the same *Jag1* gain-of-function genotype that were not induced with doxycicline. Another control group consisted of TRAMP.Tet-O-*Jag1*.Tie-2-rtTA^−^ mice administered with doxycycline to discard possible doxycycline driven effects. No differences in tumor growth dynamics or tumor vascular phenotypes were found between the two control groups (data not shown).

The loss-of-function mutant is a conditional “knock-out” where the coding region for the DSL (Delta-Serrate-Lag2) region of *Jag1* (exon 4) is flanked by loxP sites- *Jag1^lox/lox^* line [45] (B6; 129S-*Jag1^tm2Grid^*/J; The Jackson Laboratory). *Jag1^lox/+^* mice were crossed with VE-Cadherin-Cre-ER^T2^ mice [46] in order to obtain a *Jag1^lox/lox^* VE-Cadherin-Cre-ER^T2^ mouse line. *Jag1* null endothelial mutants were generated upon treatment with tamoxifen (50mg/kg daily IP for 5 days, starting one week before the experiment). One control group had the same *Jag1* loss-of-function genotype but were not induced with tamoxifen. Another control group consisted of TRAMP.*Jag1^lox/lox^* VE-Cadherin-Cre-ER^T2-^ mice administered with tamoxifen to discard possible tamoxifen specific effects. No differences in tumor growth dynamics or tumor vascular phenotypes were found between the control groups (data not shown).

### LLC subcutaneous tumor model

Lewis Lung Carcinoma (LLC) (ATCC^®^ CRL-1642^TM^) [47] cells were cultured in RPMI 1640 medium (Gibco 21875-034) supplemented with 10% foetal bovine serum (Gibco 10270-106) and 1% penicillin/streptomycin (Gibco 15140-122) in 100mm tissue culture dishes (Corning 734-1705) coated with poly-D-Lysine Hydrobromide (Sigma P7280) at 37 °C in a humidified atmosphere of 95% air and 5% CO_2_.

When cells reached subconfluence, they were detached by 5 min treatment with 0,25% trypsin-EDTA (Gibco 25200-056) and resuspended in PBS to a cell concentration of 1×10^7^/ml. For the transplant tumor model, cells (1×10^6^/mouse) were inoculated subcutaneously, in the right flank with the mouse under anaesthesia (2,5% avertin).

### Tissue preparation and immunohistochemistry

Subcutaneous tumor transplants were collected at day 14^th^ after LLC injection and, in the TRAMP model, prostates were dissected at 18 or 24 weeks of age.

For histopathological analysis, prostates were fixed in 10% buffered formalin solution for 48 h, dehydrated in alcohol, cleared in xylene, embedded in paraffin, sectioned at 3μm and stained with hematoxylin (Fluka AG Buchs SG Switzerland) and eosin Y (Sigma, St. Louis, MO). The sections were then analysed blindly by a pathologist (CP) and scored according to the literature [18]. Tumor samples from both models, were also fixed with 4% paraformaldehyde (PFA) solution at 4°C for 1h, cryoprotected in 15% sucrose, embedded in 7,5% gelatin, frozen in liquid nitrogen and cryosectioned at 10 and 20μm.

To examine vascular density and vessel maturity a rat monoclonal anti-mouse PECAM-1 (BD Pharmingen, San Jose, CA) and a mouse monoclonal anti-SMA Cy3 conjugate (Sigma Aldrich, USA), combined with a donkey anti-rat conjugated with Alexa Fluor 488 (Invitrogen, Carlsbad, CA) were used. Nuclei were counterstained with 4′, 6′-diamidino-2-phenylindole dihydrochloride hydrate (DAPI; Molecular Probes, Eugene, OR). Vascular density is equivalent to the percentage of each tumor section field occupied by a PECAM-1-positive signal (as determined by the percentage of black pixels per field after transforming the RGB images into binary files). Similarly, as a measure of vascular maturity, mural cell recruitment was assessed by quantifying the percentage of PECAM-1-positive structures lined by α-SMA-positive cells.

To assess vascular perfusion, avertin (2,5%) anesthetized mice were injected with biotin-conjugated lectin from *Lycopersicon esculentum* (100μg in 100μl of PBS; Sigma, St. Luis, MO) via caudal vein and allowed to circulate for 5 minutes before perfusing the vasculature transcardially with 4% PFA in PBS for 3 minutes. Slides were stained with rat monoclonal anti-mouse PECAM-1, followed by Alexa 594 goat anti-rat IgG (Invitrogen, Carlsbad, CA). Biotinylated lectin was visualised with Streptavidin-Alexa 488 (Invitrogen, Carlsbad, CA). Tumor perfusion area was quantified by determining the percentage of PECAM-1-positive structures that were co-localized with Alexa 488 signals.

To analyse vascular extravasation, avertin anesthetized mice were injected with 1% Evans Blue dye solution (Sigma, St. Luis, MO) via caudal vein, and perfused transcardially 5 minutes later with 4% PFA in PBS for 3 minutes. Tissue sections were stained with rat monoclonal anti-mouse PECAM-1, followed by Alexa 488 goat anti-rat IgG. Tumor vascular extravasation area was quantified by determining the tumor section field of Evans Blue red positive signal per vessel area (given by vascular density measurements).

For evaluation of hypoxic levels a rabbit anti-Hif1α antibody was used (Abcam, Cambridge, UK). Additionally, Hypoxyprobe^TM^-1 Plus Kit (Hypoxyprobe, Inc, USA) was used to detect cells with low oxygen pressure (pO2 = 10 mmHg), in paraffin embedded sections.

For quantification of cellular apoptosis and proliferation, a rabbit anti-active caspase3 (Cell signaling Technology) and an Alexa-570 conjugated mouse anti-Ki67 (eBiosciences Inc., CA, USA) antibodies were used.

For the assessment of epithelial to mesenchymal transition, an Alexa-488 conjugated mouse anti-E-cadherin and a goat polyclonal anti-Snail (Abcam, Cambridge, UK) antibodies were used, respectively.

Additional primary antibodies used were goat anti-Jagged1 (Sigma), rat anti-Vegfr-2 (Cell signalling Technology), Alexa-488 conjugated rabbit anti- NG-2 (Milipore) rabbit anti-Hey1 (Milipore), rabbit anti-N3ICD and goat anti-N4ICD (Santa Cruz Biotechnology). Additional secondary antibodies used were Alexa-647 donkey anti-goat, anti-rat and anti-rabbit (Invitrogen, Carlsbad, CA).

### Quantitative transcriptional analysis

For whole prostate analysis, tumor samples were collected at the endpoint of each experiment and snap frozen for RNA extraction (Qiagen, Hilden, Germany). For ECs and vSMCs specific analysis, samples were collected at the endpoint of each experiment and prepared for FACS sorting. ECs and mural cells were sorted directly into the lysis buffer of the RNeasy Micro Kit (Qiagen). Total RNA was isolated according to manufacturer's protocol. A total of 100 ng RNA per reaction (ECs and vSMCs) and 400 ng per reaction (whole prostate) was used to generate cDNA with the SuperScript III First Strand Synthesis Supermix Q RT-PCR Kit (Invitrogen, CA). Relative quantification real-time PCR analysis was performed as described [48] using Sybergreen Fastmix ROX dye (Qiagen). Primer pair sequences are available on request. The housekeeping gene *β-actin* was used as endogenous control.

### Flow cytometry

For flow cytometric analysis and sorting of ECs (Lin^−^ (cd45^−^ ter119^−^) cd31^+^) and mural cells (Lin^−^ (cd45^−^ ter119^−^) cd146^+^ cd31^−^) [49], prostates were collected and finely dissected into small pieces (2-4 mm). Then, the samples were digested into 1 ml solution of 1% collagenase (Sigma) and 2,4U/ml of dispase (Gibco, Life Technologies) incubation at 37°C, with agitation, for 2h30 min. DNAse I (Sigma) was added during digestion to eliminate DNA residues. After washing, digested cells were then subjected to immunostaining with anti-mouse ter-119 PE-Cy7, anti-mouse cd45 PE-Cy7 (Affymetrix, eBioscience), anti-mouse cd31 FITC and anti-mouse cd146 PE (BD Pharmingen). After washing, cells were sorted in FACS Aria III cytometer and analyzed using BD FlowJo software (Version 10.0, BD Bioscience).

For demarcating and sorting ECs and mural cells, first standard quadrant gates were set, subsequently to differentiate cd31^+^ (>10^3^ log FITC fluorescence) and cd146^+^ (>10 log PE fluorescence) cells from the Lineage negative population (≤10^2^ log PE-Cy7 fluorescence).

### Statistical analysis

All data processing (except most common prostatic lesion) was carried out using the Statistical Package for the Social Sciences software, version 17.0 (SPSS v. 17.0; Chicago, IL). Statistical analyses were performed using Mann-Whitney-Wilcoxon test and Student's t-test.

Scores of the most common histopathological prostatic lesions were analyzed with the GLM procedure of Statistical Analysis System (SAS Institute Inc. v.9.1.3 2009; Cary, USA). The analyses were carried out within group, with a linear model including the effects of time (weeks 18 and 24), endothelial *Jag1* modulation (e*Jag1*OE and e*Jag1*cKO vs. respective controls) and their interaction.

All results are presented as mean ± SEM. *P*-values < 0.05, <0.01 and <0.001 were considered significant (indicated in the figures with *) and highly significant (indicated with ** and ***), respectively.

## SUPPLEMENTARY MATERIAL FIGURES


