# Supplementary material for: Endothelial Jagged1 promotes solid tumor growth through both pro-angiogenic and angiocrine functions
Source: Oncotarget. 2015 Jun 8;6(27):24404–23. doi: 10.18632/oncotarget.4380 (PMC4695194; doi:10.18632/oncotarget.4380)
Supplement: Supplementary file 1 [file oncotarget-06-24404-s001.pdf]

Endothelial Jagged1 promotes solid tumor growth through both pro-angiogenic and angiocrine functions

Supplemental material

Extended Methods

Experimental animals- All animals were housed in ventilated propylene cages with sawdust as bedding, in a room with temperature between 22ºC and 25ºC and a 12-hours-light/12-hours-dark cycle. The mice were fed standard laboratory diet.

Immunofluorescence analysis- Immunofluorescence was performed using the following protocol: tissue slides were permeabilized in 3% H_2O_2 methanol solution for 30 min and PBS-Triton 0,1% solution 2x 10 min; blocking was performed for 1h (room temperature) either with 2% BSA + 5% Donkey serum in PBS-W 0,1%; after blocking, slides were incubated over-night at 4ºC with specific primary antibodies followed by 1h incubation at room-temperature with fluorescently-tagged specific secondary antibodies.

For PSMA staining, rabbit monoclonal anti-PSMA4 (Abcam, Cambridge, UK) was used. For pericyte coverage rabbit monoclonal anti- PDGFRβ (Cell signaling Technology) was used. Similarly to mural cell recruitment analysis, coverage was assessed by quantifying the percentage of PECAM-1-positive structures lined by pdgfr-β positive cells.

Instrument details- Fluorescent immunostained sections from tumor transplants were examined under a Leica DMRA2 fluorescence microscope with Leica HC PL Fluotar 10X, 20X and 40X/0.5 NA dry objectives (Leica, Heidelberg, Germany), captured using Photometrics CoolSNAP HQ, (Photometrics, Friedland, Denmark), and processed with Metamorph 4.6-5 (Molecular Devices, Sunnyvale, CA). Fluorescent immunostained sections from prostatic tumors, due to the tissue complexity, were obtained using a Carl Zeiss LSM 710 confocal microscope with either Zeiss 20X (Plan-Apochromat) NA 0.80 dry objective or 40X (EC Plan-Neofluor) NA 1.30 oil immersion objective, and captured using ZEN 2010 software (Carl Zeiss, Jena, Germany). Morphometric analyses were performed using the NIH ImageJ 1.37v program (NIH, Bethesda, MA, USA).

H&E stained sections were examined under a Olympus BX51 microscope with Olympus 10X/0.30 NA and 40X/0.75 NA dry objectives and captured with coupled Olympus DP21 photographic equipment (Olympus Iberia, Inc).

Suppl.Figure 1

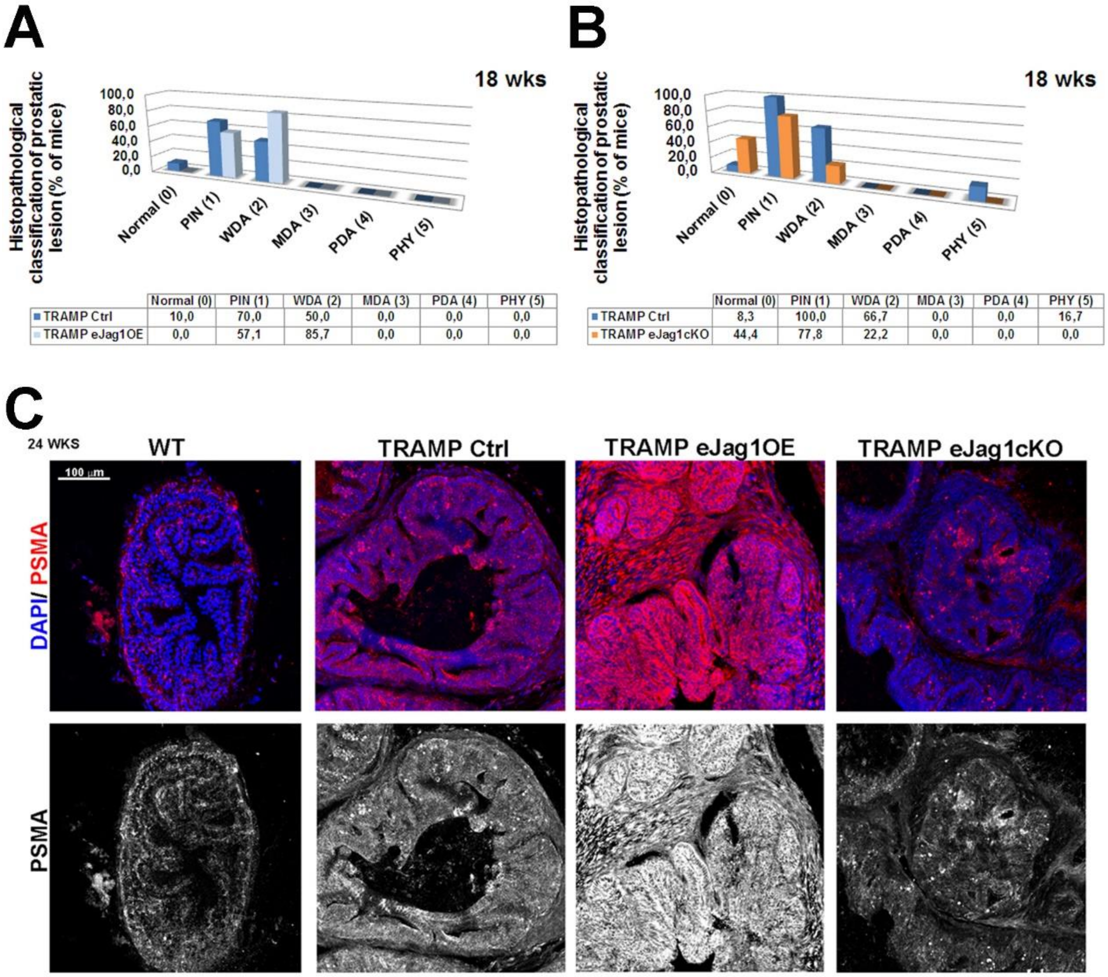

	Normal (0)	PIN (1)	WDA (2)	MDA (3)	PDA (4)	PHY (5)
TRAMP Ctrl	10,0	70,0	50,0	0,0	0,0	0,0
TRAMP eJag1OE	0,0	57,1	85,7	0,0	0,0	0,0

	Normal (0)	PIN (1)	WDA (2)	MDA (3)	PDA (4)	PHY (5)
TRAMP Ctrl	8,3	100,0	66,7	0,0	0,0	16,7
TRAMP eJag1cKO	44,4	77,8	22,2	0,0	0,0	0,0

Supplemental Figure 1. Modulation of endothelial *Jag1* in TRAMP mice

(A) and (B) Frequency distribution (% of mice) of histophatological classification of prostatic lesions at 18 weeks of age in TRAMP.e*Jag1*OE and TRAMP.e*Jag1*cKO mutants, respectively, versus controls. (C) Representative images of prostate specific membrane antigen (PSMA) (red) immunostaining demonstrating increased positive signal in TRAMP.e*Jag1*OE while decreased positive staining in TRAMP.e*Jag1*cKO mutants relative to controls (TRAMP Ctrl). DAPI (blue) stains nuclei.

Suppl. Figure 2

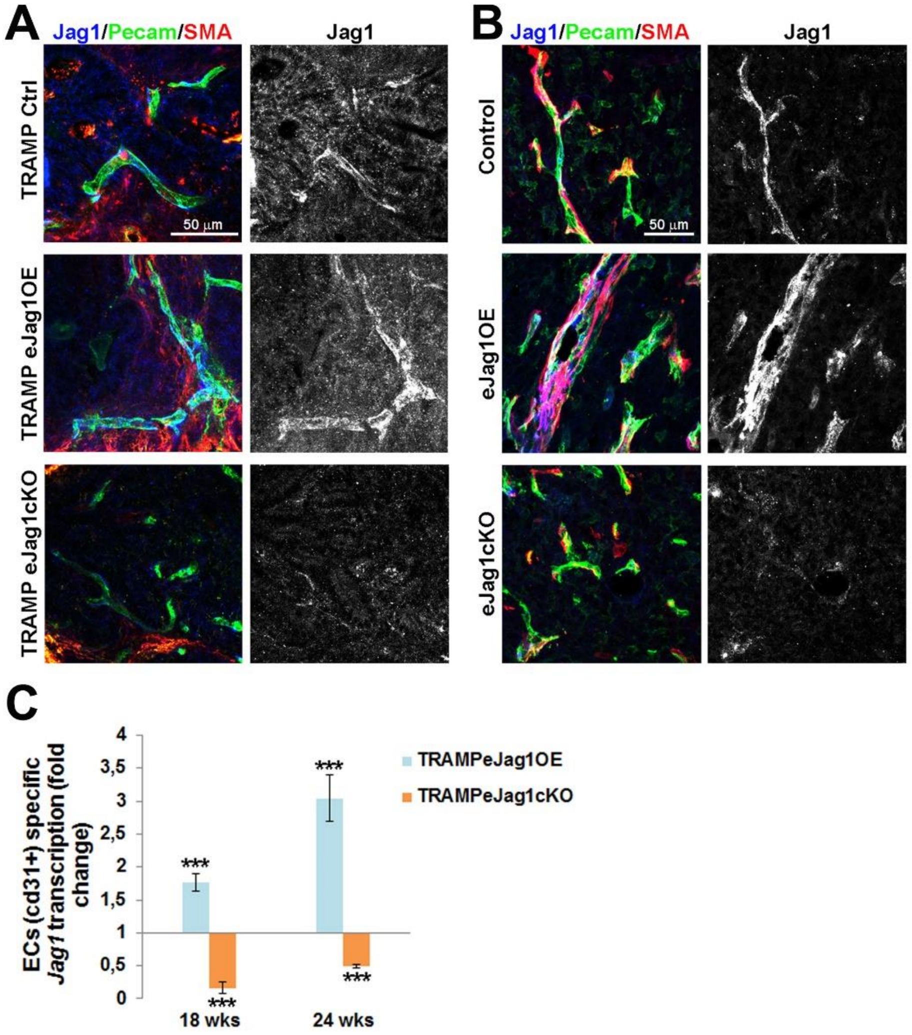

Supplemental Figure 2. Jag1 expression and transcription in endothelial specific *Jag1* mutants

(A) and (B) Representative confocal immunostaining images (40x amplification) marked for Jag1 (blue), Pecam (green), and SMA (red) to evaluate specific modulation of endothelial Jag1 in TRAMP and LLC tumor samples, respectively. DAPI (blue) stains nuclei. (C) ECs specific *Jag1* transcription analysis in TRAMP.e*Jag1* prostates. Error bars represent SEM; ** represents p<0.01; *** represents p<0.001.

Suppl. Figure 3

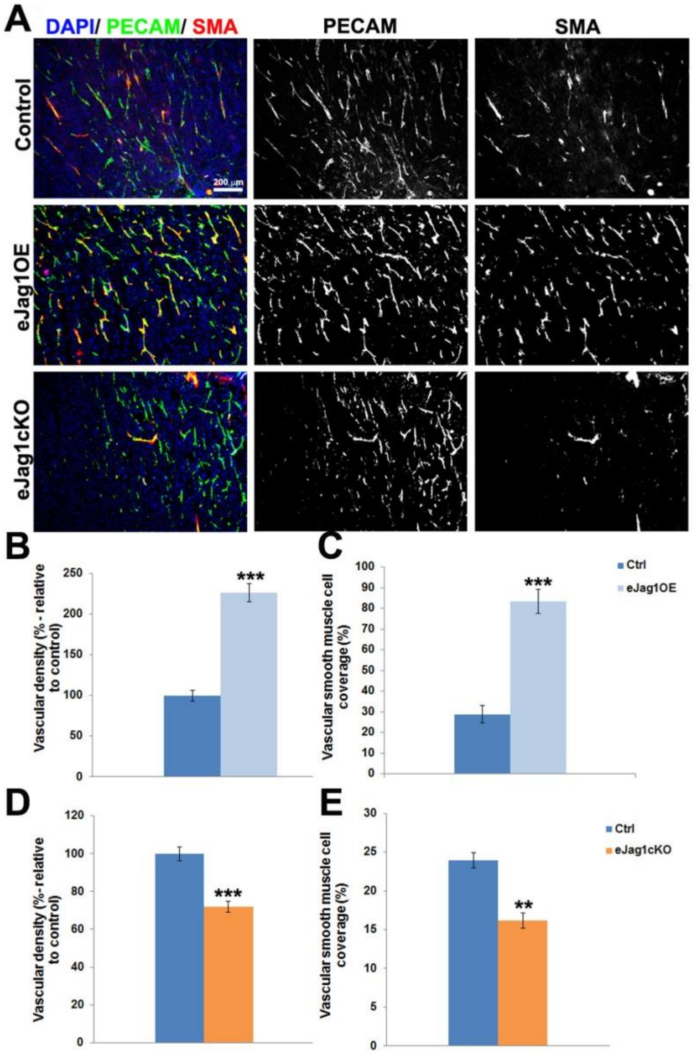

Supplemental Figure 3. **LLC xenograft tumor vascular phenotype in endothelial specific *Jag1* mutants**

(A) Representative immunostaining images (10x amplification) marked for PECAM-1 (green) and SMA (red), to evaluate vascular density and vSMC of xenograft samples. (B) Percentage of vascular density (relative to control=100%) is increased in endothelial *Jag1* over-expression mutants as shown by PECAM-1 labeling. (C) Percentage of vascular smooth muscle coverage, showing increased levels of SMA on *eJag1OE* mutant vasculature, relative to controls. (D) Percentage of vascular density (relative to control=100%) is decreased in endothelial *Jag1* knock-out mutants. (E) Percentage of vascular smooth muscle coverage, showing decreased levels of

SMA on *eJag1c*KO mutant vasculature, relative to controls. DAPI (blue) stains nuclei. Error bars represent SEM; ** represents p<0.01; *** represents p<0.001.

Suppl. Figure 4

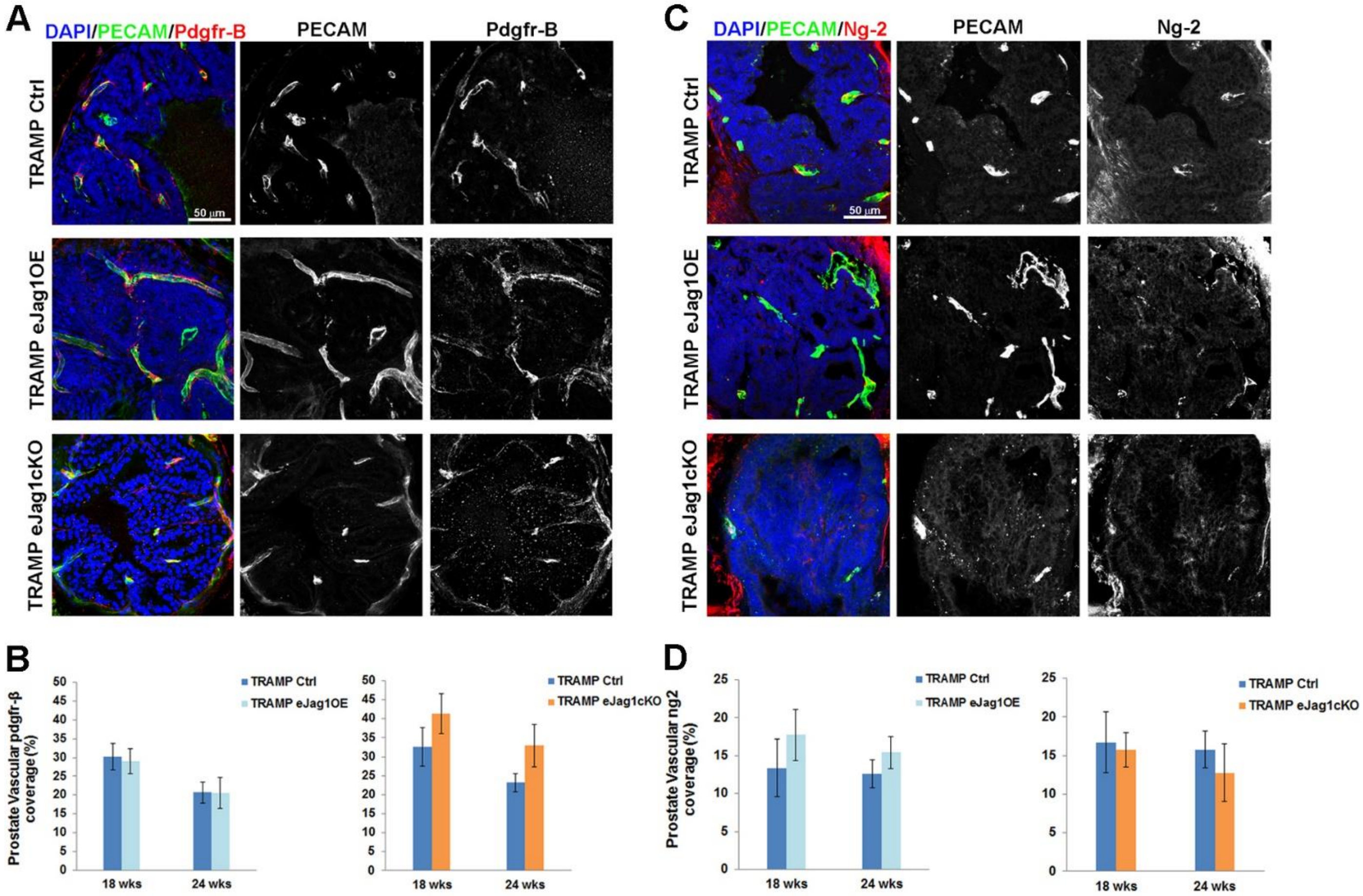

Supplemental Figure 4. Prostate tumor endothelial pericyte coverage in TRAMP endothelial-specific *Jag1* mutants

(A) Representative confocal immunostaining images (40x amplification) marked for PECAM-1 (green) and Pdgfr-β (red), to evaluate pericyte vascular coverage of prostate samples. (B) Percentage of prostate vascular Pdgfr-β coverage in TRAMP.e*Jag1*OE and KO mutants showing no significant difference from the controls. (C) Representative confocal immunostaining images (40x amplification) marked for PECAM-1 (green) and Ng-2 (red), to evaluate pericyte vascular coverage of prostate samples. (D) Percentage of prostate vascular Ng-2 coverage in TRAMP.e*Jag1*OE and KO mutants showing no significant difference from the controls.

Suppl. Figure 5

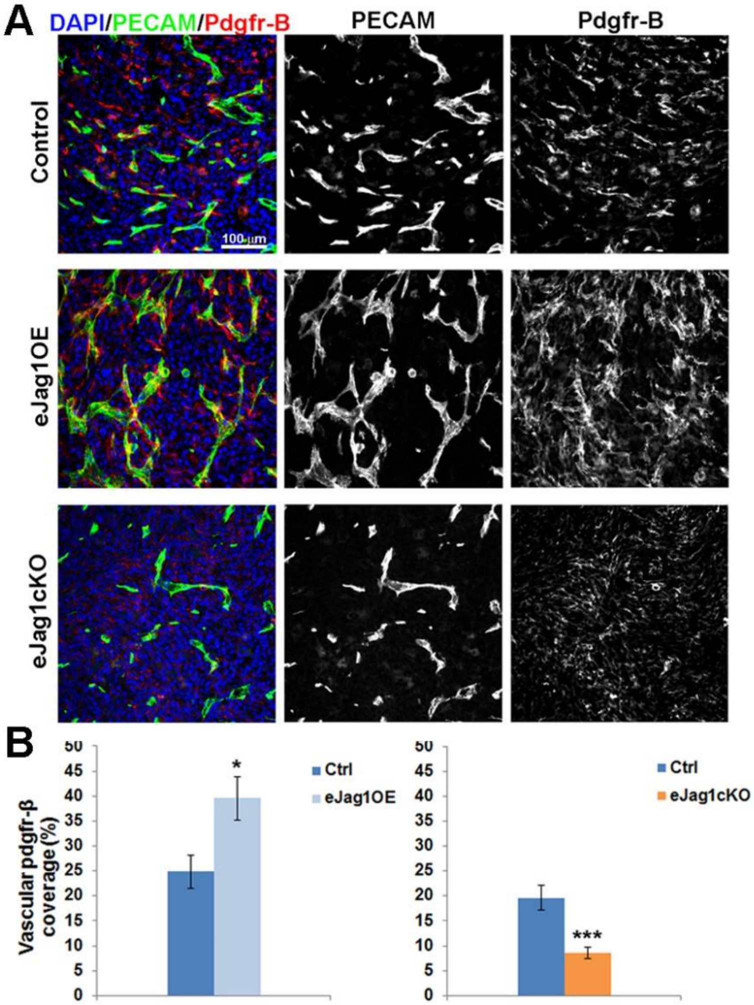

Supplemental Figure 5. LLC xenograft tumor endothelial pericyte coverage in endothelial-specific *Jag1* mutants

(A) Representative immunostaining images (20x amplification) marked for PECAM-1 (green) and Pdgfr-β (red), to evaluate pericyte vascular coverage of LLCs tumor samples. (B) Percentage of vascular Pdgfr-β coverage in e*Jag1*OE and KO mutants showing increased and decreased coverage relative to the controls, respectively. DAPI (blue) stains nuclei. DAPI (blue) stains nuclei. Error bars represent SEM; ** represents $p<0.01$; *** represents $p<0.001$.

Suppl. Figure 6

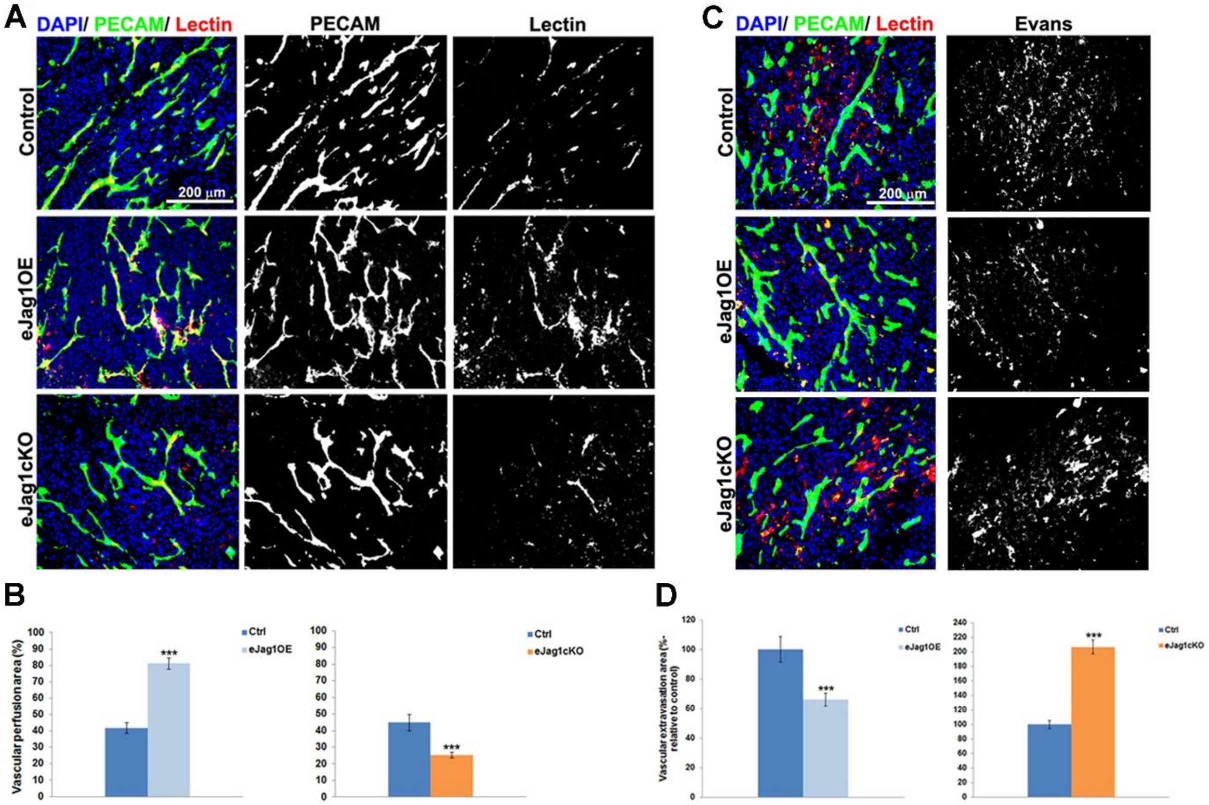

Supplemental Figure 6. LLC xenograft tumor vascular perfusion and extravasation in endothelial specific *Jag1* mutants

(A) Lectin (red) and PECAM-1 (green) immunostaining (20x amplification) of e*Jag1*OE and e*Jag1*cKO mutants xenografts, to evaluate the co-localization of both signals, indicative of vessel perfusion. (B) Percentage of perfused area in the total vascular area (given by vascular density measurements) showing increased and decreased lectin labeling in the endothelial *Jag1* over-expression and loss-of-function vasculature, respectively. (C) Evans' Blue (red) and PECAM-1 (green) immunostaining (20x amplification) images showing the extravasation areas. (D) Percentage of vascular extravasation area in the total vascular area, showing decreased Evans' Blue staining in *eJag1*OE, while increased in e*Jag1*cKO mutants. DAPI (blue) stains nuclei. Error bars represent SEM; *** represents p<0.001.

Suppl. Figure 7

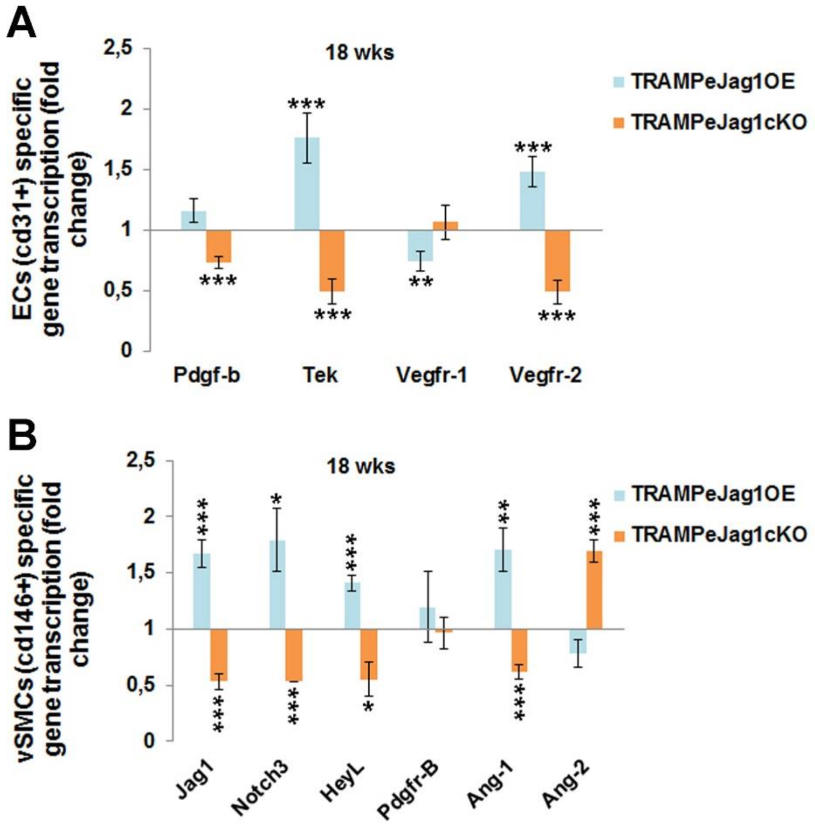

Supplemental Figure 7. Transcription profile of angiocrine factors by endothelial and perivascular tumor associated cells in TRAMP endothelial-specific *Jag1* mutants at 18 weeks of age

RNA was isolated from prostates collected at the end-point, and gene transcript analysis was performed by quantitative real-time RT-PCR for genes involved in angiogenesis. (A) ECs specific relative gene transcription. (B) vSMCs specific relative gene transcription. Gene transcript levels were normalized to PECAM-1 mRNA levels, and the house-keeping gene β-actin was used as endogenous control. Blue bars represent the gene expression levels of samples collected from *eJag1*OE mutants, and orange bars the gene expression levels from e*Jag1*cKO mutants, relative to the respective controls. Error bars represent SEM; * represents p<0.05; ** represents p<0.01; *** represents p<0.001.

Suppl. Figure 8

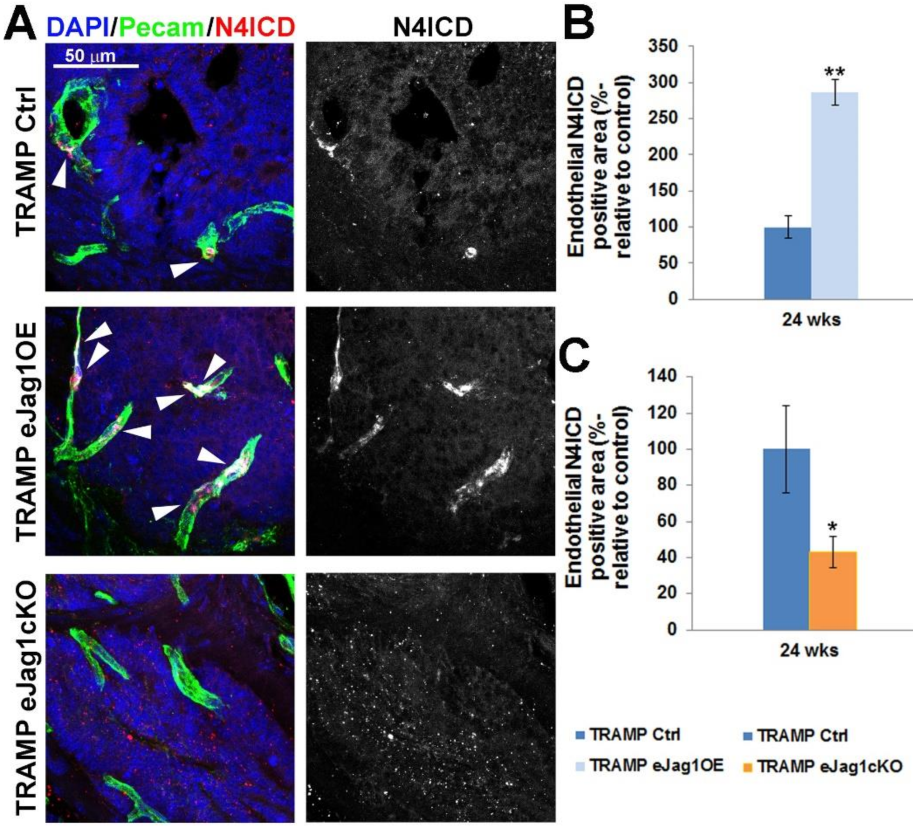

Supplemental Figure 8. Immunostaining for Notch4 intra-cellular domains (N4ICD) in TRAMP.*eJag1OE* and *eJag1cKO* mutants.

(A) High-magnification confocal immunostaining images (40x amplification) of PECAM-1 (green) and N4ICD (red) in TRAMP.e*Jag1* mutants. White arrows indicate N4ICD expression in endothelial cells. (B) Percentage of endothelial N4ICD-positive area (relative to control=100%) showing increased N4ICD in e*Jag1*OE mutant ECs. (C) Percentage of endothelial N4ICD positive area (relative to control-100%), showing the decreased co-localization of N4 activated form with ECs (white arrows) in e*Jag1*cKO mutants. DAPI (blue) stains nuclei. Error bars represent SEM; * represents p<0.05; ** represents p<0.01; *** represents p<0.001.
